# Methods to Scale Down Graphene Oxide Size and Size Implication in Anti-cancer Applications

**DOI:** 10.3389/fbioe.2020.613280

**Published:** 2020-12-23

**Authors:** Immacolata Tufano, Raffaele Vecchione, Paolo Antonio Netti

**Affiliations:** ^1^Center for Advanced Biomaterials for HealthCare@CRIB, Istituto Italiano di Tecnologia, Naples, Italy; ^2^Department of Chemical, Materials and Industrial Production Engineering, University of Naples Federico II, Naples, Italy; ^3^Interdisciplinary Research Center of Biomaterials, University of Naples Federico II, Naples, Italy

**Keywords:** nanomedicine, 2D nanomaterials, graphene oxide, anticancer treatment, theranostic tool, photoacoustics

## Abstract

Despite considerable progress in the comprehension of the mechanisms involved in the origin and development of cancer, with improved diagnosis and treatment, this disease remains a major public health challenge with a considerable impact on the social and economic system, as well as on the individual. One way to improve effectiveness and reduce side effects is to consider responsive stimuli delivery systems that provide tailor-made release profiles with excellent spatial and temporal control. 2D nanomaterials possess special physicochemical properties (e.g., light, ultrasonic and magnetic responses) and biological behaviors such as endocytosis, biodistribution, biodegradation, and excretory pathways, which lead to their use in various biomedical applications. In particular, among 2D nanomaterials, graphene and its derivatives, namely graphene oxide (GO) nanomaterials, have attracted enormous attention in cancer diagnosis and therapy because they combine, in a unique material, extremely small size, NIR absorption, delocalized electrons, extremely high surface area, and versatile surface functionality. Taking into account the fundamental role played by GO size, in this review, we summarize the main methods employed to reduce and homogenize in nanometric scale the lateral dimensions of graphene oxide produced by chemical exfoliation of graphite, as well as post-synthesis separation techniques to uniform the size. We also discuss the implication of the small size in cancer treatment by exploiting GO nanocarriers as an effective theranostic tool.

## Introduction

**FIGURE 1:**
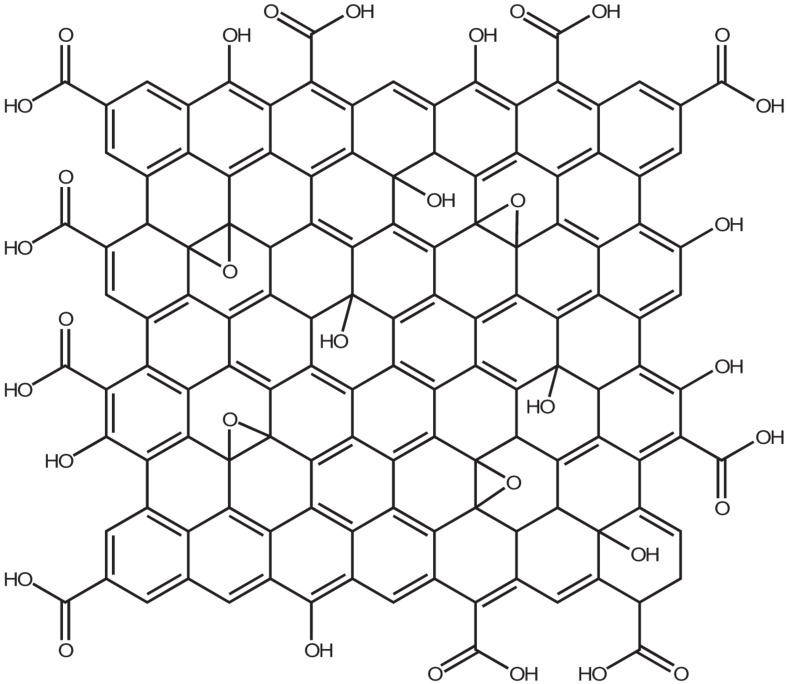
Chemical structure of Graphene Oxide based on the Lerf-Klinowski model.

In accordance with the latest report of the International Agency for Research on Cancer, the incidence of cancer is increasing worldwide (Bray et al., 2018). Diagnosis has progressively increased from 14.8 million in 2014 to 18.1 million in 2018 (of which 23.4% in Europe) and 29.5 million are expected in 2040. Global cancer mortality has increased from 8.2 million in 2014 to 9.6 million in 2018. Patients surviving five years after diagnosis are currently 43.8 million. Despite considerable progress in diagnosis and treatment, this remains one of the most critical diseases with an enormous impact on the social and economic system (Torre et al., 2015). Current cancer treatment requires surgical resection (for solid tumors), followed by radiation therapy, chemotherapy, or immunotherapy to kill the remaining cancer cells. The term cancer is used to define a series of critical diseases associated with replication and uncontrolled cellular spread. The abnormal proliferation of cells develops a tumor malformation characterized by a heterogeneous and complex microenvironment, which includes blood vessels, immune cells, and signaling molecules. This dynamic microenvironment strongly influences the growth and evolution of the tumor and the success of therapies. The impressive progress achieved in recent decades on the etiology of cancer has not even translated into tangible progress in therapy (Srinivasan et al., 2016). It is well known that most conventional chemotherapeutic drugs show unfavorable chemical-physical and pharmacological properties such as low aqueous solubility, irritating nature, lack of stability, rapid metabolism, and non-selective drug distribution (Iwamoto, 2013). These properties cause several adverse effects, including less therapeutic activity, dose-limiting side effects, low bioavailability of the anticancer drug at the site of action as well as high organ toxicity limiting the maximum tolerated dose and patient quality of life. Besides, resistance to many of the most active cytotoxic agents used in cancer therapy can occur in many tumors. Some tumors, initially responsive, recur and become resistant not only to the initial therapeutic agents but also to other drugs not used for treatment (Tomida and Tsuruo, 2002). This phenomenon is known as multidrug resistance (MDR) and is one of the main causes of failure in chemotherapeutic treatments. Therefore, it is necessary to develop highly efficient therapeutic systems able to overcome biological barriers, selectively distinguish cancerous tissues from healthy ones and react “intelligently” to the heterogeneous and complex tumor microenvironment for the on-demand release of therapeutic agents in the optimal dosage range (Sun et al., 2014). As early as 2006, the National Cancer Institute recognized nanotechnology as the science that can effectively change the basis for the diagnosis, treatment, and prevention of cancer, allowing the study and treatment of this disease at a molecular scale, in real-time and during the early stages of the process. Although particles between 10 and 100 nm are known since ancient times as components of pigments and dyes, the concept of nanotechnology as a potential manipulation of matter on the atomic scale is quite recent (Thrall, 2004). The application of nanotechnology to disease treatment, diagnosis, monitoring, and control of biological systems is referred to as “Nanomedicine.” The success of nanotechnology in the healthcare field is due to the possibility of working at the same scale as many biological processes, cellular mechanisms, and organic molecules (Liu et al., 2007). The traditional application of nanotechnology in cancer therapy is to improve pharmacokinetics and reduce the side effects of chemotherapy through nanocarriers that target selectively and deliver anti-cancer drugs to tumor tissues. The nanocarriers used for drug delivery are manufactured from both soft (organic and polymeric) and hard (inorganic) materials assembled in different architectures such as polymeric micelles, nanoparticles, liposomes, and dendrimers, which share the dimensional characteristic of the nanometric scale. The active cargo can be easily encapsulated or covalently bonded with the nanocarriers exploiting the chemical-physical properties of the materials that compose them (Fleige et al., 2012). The nanostructured transport systems are originally designed to increase cellular uptake and accumulation due to their nanometric size. One of the unique features of almost all solid tumors is “leaky” tumor vascularization and compromised lymphatic drainage. As previously described, a growing tumor mass generates a network of abnormal blood vessels (angiogenesis) for increasing oxygen and nutrients supply. This feature, combined with the poor lymphatic clearance typical of tumor masses, allows particles between 10 and 100 nm in size to passively penetrate through the pores of blood vessel walls and accumulate preferentially in tumor sites rather than in other healthy tissues. This effect, known as enhanced permeability and retention, (EPR) has shown promising results compared to standard therapies in terms of reduced toxicity in healthy tissues and increased drug concentration at the target site. Over the past 20 years, a variety of nano-carriers such as liposomes, micelles, albumin nanoparticles and polymeric conjugates have been approved for the treatment of various tumors (Ventola, 2017). However, the nanocarrier exploiting the EPR effect must necessarily have a long circulation time in the blood to arrive at the target area. One of the approaches commonly used to increase the efficiency of drug transport to cells and cancerous tissues is to modify the surface of nanocarriers with water-soluble polymers such as polyethylene glycol (PEG). Because of their hydrophilic nature, PEG chains create a hydrated cloud that protects the surface from aggregation, opsonization, and phagocytosis, increasing blood circulation time (Suk et al., 2016). Doxil^®^, PEG-coated liposomal doxorubicin was approved by the US FDA in 1995 and is used for the treatment of breast cancer through the accumulation of passive cancer. However, the passive targeting of drug carrier systems through the EPR effect has some limitations. First, the EPR effect is highly biologically dependent on the degree of vascularization and angiogenesis of the tumor. Also, the high interstitial pressure in the central area of the tumor not only inhibits the delivery of the drug by convection but also compresses new blood vessels. As a result, blood is led away from the center of the tumor to the periphery (Attia et al., 2019). An ideal nanocarrier should simultaneously show a high accumulation in the tumor and cellular internalization after achieving tumor tissue. However, the EPR effect does not enhance the uptake of nanoparticles into the cells. For this reason, next-generation therapeutic nanoparticles have an active targeting mechanism. Active targeting is usually performed by binding a variety of specific ligands to the surface of the nanocarrier that can recognize specific surface molecules that are overexpressed by tumor cells but not present in normal cells. Through this mechanism, it is possible to increase the affinity of the nanocarrier for the surface of cancer cells or tumor tissue and thus significantly increase the amount of drug delivered to the target cell compared to the free drug or passive targeting nanosystems (Qiao et al., 2019). The stochastic nature of ligand-receptor interactions in active targeting and the lack of cell, tissue, and organ specificity of the laws governing the spread of the nanocarrier within the body in passive targeting, result in these processes being poorly applied in the clinic and suggest the need for more efficient delivery strategies (Mura et al., 2013). One way to improve effectiveness and reduce side effects is to consider responsive stimuli delivery systems that provide tailor-made release profiles with excellent spatial and temporal control. Compared to traditional nanocarriers, responsive stimuli delivery systems are designed to improve solubility, bioavailability and to prolong the blood circulation time, besides, they can be chemically optimized to achieve selective drug release at desired action sites, which can enable them to bypass physiological or pathological barriers and achieve higher therapeutic efficacy (Vijayakameswara Rao et al., 2018). Commonly this approach has been realized with biocompatible materials properly engineered to undergo a specific protonation, a hydrolytic rupture, a conformational change in response to a specific stimulus. The investigated stimuli include endogenous stimuli (e.g., reactive oxygen species (ROS), redox, pH, and enzymes) and exogenous stimuli (e.g., light, temperature, magnetic field, and ultrasound). Another aspect to consider in designing an effective therapy plan is the real-time monitoring of the therapy. With conventional treatments, diagnosis, therapy, and evaluation of the effect of the therapy are independent processes. This prolongs healing times and increases the suffering of cancer patients, especially when considering that contrast agents used in classical imaging techniques are not free of side effects. To overcome these obstacles, a promising clinical application consists of systems able to combine drug and diagnostic agents on the same nanocarriers to detect disease status and also provide therapeutic agents to target sites with real-time monitoring of pharmacokinetics, distribution and accumulation of drugs in tumors, leading to effective tumor inhibition as soon as possible (Zhao et al., 2018). Nano-systems that can integrate imaging and therapy are referred to as nano-theranostics. If the materials selected for nano-theranostic systems are responsive to electromagnetic, sound, or thermal fields, it is also possible to combine chemotherapy and imaging with other treatments. One of the most studied topics in recent years is the combination of chemotherapy with photothermal therapy (PTT) since photothermal therapy is a minimally invasive and potentially effective treatment. Photothermal therapy is a physical-chemical therapy for the treatment of cancer that employs optical radiation in the near-infrared (NIR) wavelength range (700-2000 nm). When a laser is focused on a tissue, the photons are absorbed by the intercellular and intracellular areas and the energy of the photons is converted into heat. As a result, the temperature of the tissues increases, leading to the death of cells and tissues (Lo, 2017). The local increase in temperature due to radiation not only causes the ablation of tumors but improves the permeability of the cell membrane, making the accumulation of nanoparticles in tumor cells more effectively and hinders the repair of DNA damage caused by anticancer drugs in tumor cells, increasing the effects of chemotherapy agents (Park et al., 2010). In addition, NIR radiation used, also known as “optical window” or “therapeutic window,” is the wavelength range that has the greatest depth of penetration into tissues. The most widely accepted NIR photothermal agents include fluorescent dyes, two-dimensional inorganic materials (e.g., carbon nanotubes, graphene oxide, and gold materials), and polydopamine (Sakudo, 2016). Ultra-thin two-dimensional (2D) nanomaterials are a large class of nanomaterials with sheetlike structures, lateral dimensions greater than 100 nm, and thickness less than 5 nm (Tan et al., 2017). This class of materials is in continuous evolution and includes nanomaterials of different chemical compositions and crystalline structures. Generally speaking, ultra-thin 2D nanomaterials are distinguished as layered and non-layered 2D nanomaterials. In layered nanomaterials, the atoms in each layer are connected by strong chemical bonds, while the layers stack together to form bulk crystals through weak van der Waals interactions. In contrast, non-layered nanomaterials crystallize in three dimensions through atomic or chemical bonds to form bulk crystals. The study of 2D nanomaterials was first reported when Novoselov et al. (2004) succeeded in exfoliating graphene from graphite (Novoselov et al., 2004), and since then research on ultra-thin two-dimensional nanomaterials has grown exponentially in the fields of condensed matter physics, materials science, chemistry, and nanotechnology. The unique characteristics of 2D nanomaterials have raised important and exciting questions about their interactions with biological systems. Being the thinnest materials, 2D nanomaterials have the highest specific surface areas among all known materials, which means that they can efficiently load and deliver therapeutic agents. Also, the planar nanostructure gives these nanomaterials special physicochemical properties (e.g., light, ultrasonic and magnetic responses) and biological behaviors such as endocytosis, biodistribution, biodegradation, and excretory pathways, which lead to their use in various biomedical applications (Chimene et al., 2015; Hu et al., 2019). Among 2D nanomaterials, graphene, and its derivatives have attracted enormous attention in cancer diagnosis and therapy because they combine, in a unique material, extremely small size, NIR absorption, delocalized electrons, extremely high surface area, and versatile surface functionality. Graphene is an allotropic form of carbon defined as a single layer (monolayer) of carbon atoms sp^2^-bounded, which are densely packed in a hexagonal honeycomb lattice (Suryanto., 2017). Since its recent discovery, this material has attracted enormous attention for its unique and, sometimes surprising, properties. Graphene is the thinnest and lightest compound known, it has a maximum tensile strength that is hundreds of times higher than steel; the electronic mobility exhibited by graphene even at room temperature, makes it an excellent heat conductor and also the best conductor of electricity and it has a great ability of optical transmittance (Rajakumar et al., 2020). In the original study of Novoselov et al. (2004) single or a few layers of pristine graphene were obtained with the “scotch tape” method of mechanical exfoliation of the graphite using adhesive tape. However, this method is not scalable, and therefore other approaches have been proposed, including chemical vapor deposition (Obraztsov, 2009), arc discharge (Subrahmanyam et al., 2009), and epitaxial growth on *SiC* (Camara et al., 2008). To date, the most widely used method when large scale graphene production is required is the wet chemical exfoliation of graphite (Eigler et al., 2013). This method involves the liquid-phase exfoliation of graphite which is composed of layers of graphene assembled parallel to each other and linked together by Van der Waals interactions. To break the interactions that hold together the graphene layers in the graphite, intense oxidation of the aromatic system is necessary. The oxidation generates an intermediate, known as graphite oxide with a high density of oxygenated functional groups and which is then transformed into reduced graphene oxide by chemical or electrochemical reduction. Graphite oxide, obtained from the oxidation of graphite, can be exfoliated in solution to form graphene oxide (monolayer) (GO), or partially exfoliated to form few-layers graphene oxide (Bianco et al., 2013). GO, initially considered as an intermediate of one of the graphene production processes, has become a material that can be considered both for fundamental research and for its potential applications. The simple, scalable, and economical production process, coupled with the peculiar chemical-physical characteristics, make GO one of the most promising nanomaterials in several fields and, notably, in the cross-section of nanotechnology and biotechnology. GO is a single or a few-layer material with a high oxygen content, typically characterized by atomic C/O ratios below 3.0 and generally closer to 2.0. Unlike the perfectly ordered crystalline structure of graphene, GO has a two-dimensional structure in which crystalline regions and regions with amorphous defects of sp^3^ (Srinivasan et al., 2016) hybridized carbons and functional groups containing oxygen, coexist (He et al., 1998; Figure 1). The different oxygenated functions located on one or both sides of the GO sheet, make this material soluble and processable in water and many organic solvents and make the surface of GO very versatile for functionalization or chemical changes to finely modify its properties or to increase biocompatibility (Singh et al., 2018).

The aromatic structure instead allows non-covalent interaction with π conjugated molecules and confers to the GO the ability to absorb light in the range of NIR (700-900 nm). This property is particularly interesting when considering cellular hyperthermia in the treatment of tumors as a minimally invasive alternative to surgery (Gonçalves et al., 2013). Furthermore, functionalized graphene oxide and nanocomposites based on GO have interesting optical and magnetic properties and can be employed as contrast agents for various biological imaging modalities including fluorescence imaging, photoacoustic imaging, and magnetic resonance imaging (Yang et al., 2013). The era of GO in cancer therapy started in 2008 with pioneering Dai’s group study. They demonstrated that polyethylene glycol-functionalized nanographene oxide (NGO-PEG) was able to efficiently complex water-insoluble aromatic drug molecules via non-covalent Van der Waals interactions. This new nanocarrier showed *in vitro* cellular uptake and killing potential for some cancer cell lines (Liu et al., 2008). Subsequently, GO was conjugated with biopolymers (Mirzaie et al., 2019), biomolecules (Li R. et al., 2020), metals (Kordi et al., 2019; Cobos et al., 2020), and metal oxides (Luo Y. et al., 2019; Pramanik et al., 2019) to create intelligent nanoplatforms able to respond at every stage of the cancer treatment process, from targeting to imaging and therapy. Recently Burnett et al. (2020) considered, for the first time, GO not as a platform for drug transport or photothermal therapy, but as the therapy itself on human osteosarcoma cancer cells. The authors aimed to evaluate the toxicity of GO on osteosarcoma *in vitro* by determining the production of reactive oxygen species (ROS) and the rate of apoptosis in normal osteoblast cell line and human osteosarcoma cell lines. In the latter case, they used the CRISPR-Cas9 technique, a molecular scissor, to remove the insulin growth factor 1 (IGF_1_) and its binding protein (IGFBP_3_) involved in the tumorigenesis. Their results showed a significantly higher rate of apoptosis and ROS generation in the osteosarcoma cells than in normal osteoblasts, especially in cells in which IGF_1_ and IGFBP_3_ were knocked out. Although it seems that GO applications in the field of cancer therapy are only limited by the creativity of scientists, to date, no biomedical nano-material based on GO has been successfully translated into clinical use in patients. This is partly due to the relatively recent application of GO in the biomedical field, but, above all, it depends on the fact that data on the biocompatibility and cytotoxic potential of graphite oxide are controversial and not yet complete. In a systematic study on the toxicity of GO *in vitro*, Chang et al. (2011) investigate the effect of GO on the morphology, viability, mortality, and membrane integrity of human lung carcinoma epithelial cell line. Their results suggest that GO has no obvious toxicity on cell lines even at high doses, but GO causes dose-dependent oxidative stress which induces a slight decrease in cell viability at the dose of 200 μg/mL (Chang et al., 2011). The overproduction of oxygen reactive species (ROS) is a known and typical toxicological mechanism of nanoparticles also of a different nature than carbon nanoparticles and has been confirmed for GO also on other cell lines, e.g., on murine lung epithelial cell lines, without any influence on viability and cell proliferation even at relatively high doses (Bengtson et al., 2016). Wang K. et al. (2011) demonstrated instead a dose-dependent toxicity of GO both *in vitro* and *in vivo*. In particular, human fibroblast cells internalize graphene oxide nanosheets predominantly in the cytoplasm, and even at 50 μg/mL doses cause apparent toxicity in terms of decreased cell survival rate, cell-floating activation and cell apoptosis. Similar serious results were found after intravenous administration in mice. GO remains in the bloodstream for a long time and mainly accumulates in the lungs, liver, and spleen. The lungs are the most affected organs and the formation of epithelioid granulomas and interstitial inflammation is observed as the dose of GO increases (Wang K. et al., 2011). Radiotracking techniques to determine the distribution of GO in mice confirm the high absorption and long-term retention of GO in the lungs, kidneys, and spleen, with less accumulation in the brain, heart, and bones. In addition, GO showed good compatibility with red blood cells (Zhang X. et al., 2011). Liu Y. et al. (2013) also highlighted the mutagenic potential of GO *in vitro* and *in vivo*. At molecular level, GO interacts with genomic DNA and interferes with DNA replication, this interaction is very rapid and reaches its maximum effect after two hours of treatment with GO at a concentration of 600 μg/mL. The authors attribute this mutagenic effect to the structural similarity between the highly planar graphene oxide nanosheets and planar aromatic DNA intercalators such as ethidium bromide or proflamine. Exactly like the DNA intercalators, GO could insert between the double helix base pairs and interfere with the flow of genetic information. To overcome this limitation, GO is usually covalently functionalized with hydrophilic polymers as polyethylene glycol. GO Pegylation can profoundly change GO cytotoxicity by attenuating the hydrophobic interactions between graphene or GO with cells and tissues (Zhang et al., 2012). One of the main causes of the controversy about the cytotoxicity of GO is undoubtedly the inhomogeneity of the material. GO is a heterogeneous material consisting of sheets with different sizes, number of layers, degree of oxidation, and chemical surface. Moreover, most of the synthetic methods used for the production of GO, although simple, low cost, and scalable, lack reproducibility as they require a long and tedious work-up that can heavily affect the chemical-physical characteristics of the material obtained. The first problem to be addressed in the design of GO-based theranostic nanoplatforms is to obtain a starting material with suitable and homogeneous dimensions. GO has size-dependent properties. First, the colloidal stability of GO sheets in aqueous solution and biological media is size-dependent. Nanometer-scale GO sheets form more stable colloidal dispersions due to the high density of charge resulting from the ionized -COOH groups at their edges (Kim et al., 2010). It is well known that the chemical-physical properties of materials, particularly their size, may regulate the cellular response to them. The size of the material impacts cellular uptake, renal clearance, transport to blood or brain barriers, and even partly the biological or toxicological effects induced by the material (Salatin et al., 2015). Graphene materials have sizes ranging from 10 nm, the size of some proteins, to more than 20 μm, larger than many cells. Large sheets can therefore adhere to the plasma membrane and spread into the cells, while small sheets can be internalized via one or more receptor-mediated endocytosis mechanisms (Sanchez et al., 2012). The mechanism of nanoparticle internalization in cells also depends on the type of cell. Yue et al. (2012) exploited the intrinsic photoluminescence of GO in NIR to study the internalization of GO samples with lateral dimensions of 350 nm and 2 μm respectively in phagocytes (e.g., Macrophages) and non-phagocytes (e.g., Endothelial cells and cancer cells) cells. Their results showed that GO internalization in all non-phagocytes cells is negligible, which is possibly due to strong electrostatic repulsions between GO and cell surface (negatively charged). In comparison to the small GO signal in non-phagocytic cells, apparent fluorescence increases are observed in phagocytic cells, indicating a high macrophage uptake potential (Yue et al., 2012). Moreover, the saturated absorption of the two GO sheets with different sizes is similar, which means that GO, unlike traditional spherical or cylindrical particles, is internalized by macrophages in a way that is independent of size (Mendes et al., 2015). In contrast, the inflammatory response in cells and animals is strongly dependent on size and GO samples between 750 and 1300 nm induce increased production of inflammatory cytokines both *in vitro* and *in vivo* compared to GO samples between 50 and 350 nm (Ma et al., 2015). As we pointed out earlier, the tumor microenvironment allows the passive accumulation of nanoparticles via the enhanced permeability and retention (EPR) effect, which essentially relies on the size of the nanoparticles. Using single proton emission computed tomography (SPECT) imaging with (Deb et al., 2018). ^125^I-radiolabeling, Cai et al. discovered that sub-50 nm is the favored size range for successful PEGylated GO tumor accumulation *in vivo* through the EPR effect (Cao et al., 2016). In particular (Deb et al., 2018), I Pegylated nano GO sheets with size less than 50 nm injected intravenously in nude mice with 4T1 tumors, displayed intense and uniform radioactive signals of the whole tumor region within 6 h after injection. The signal remained stable after 24 and 48 h and could be observed even after 96 h for the EPR effect. In contrast, the signal emitted by GO-sheets larger than 50 nm is very low even 1 h after injection and decreases rapidly, getting negligible after 6 h after injection. In addition, the distribution pattern of the two materials in healthy tissue is very similar, the liver was the organ with the greatest accumulation of both, although GO with size below 50 nm was eliminated faster than the one with a larger size. The emission of photoluminescence is also size-dependent and is attributed both to the increase in the energy gap due to the rise in oxygenated functional groups and to the nanostructure’s electronic structure (Hens et al., 2012). In nanomedicine applications, therefore, the synthesis of GO with precise dimensions and narrow size distribution is crucial. Unfortunately, GO sheets are often prepared by chemical exfoliation of graphite and the lateral dimensions of the GO prepared by this convenient method are very polydisperse in the range from tens of nanometers to a hundreds of micrometers. Therefore, it is important to design methods during or post-synthesis to satisfy the above requirements. In this review, we highlight how the size of GO sheets can be controlled and optimized in the nanometric range that is appropriate for biological applications. Although graphene materials with dimensions below 10 nm are successfully obtained by bottom-up approaches from small molecules by microwave irradiation, hydrothermal, and pyrolysis method (Gayen et al., 2019), we focus much of the review on the size control strategies for GO obtained with the simpler and more cost effective top-down graphite oxidation methodologies. First, the general methods of GO synthesis are presented. In the following sections, we describe different strategies to control the size of GO nanoparticles that we distinguish as direct controllable synthesis and post-synthesis separation. In the last section, we will emphasize the applications of nano-graphene oxide (nGO) based platforms in cancer therapy. Nanocomposites in which GO is used in combination with inorganic particles (gold, iron oxide nanoparticles) are not described in this review.

## Synthetic Approaches

**FIGURE 2:**
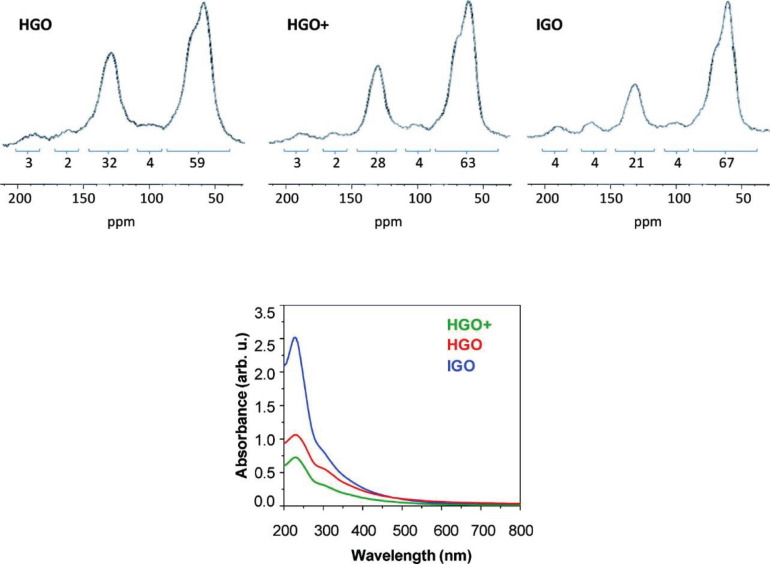
Solid-State ^13^C NMR (50.3 MHz) spectra of GO produced with Hummer method (HGO), modified Hummer method (HGO+), and GO produced with the Improved Hummer method (IGO). UV/vis spectra recorded in aqueous solutions at 0.05 mg/mL of HGO, HGO +, and IGO. Reproduced with permission from Marcano, D. C. et al. Improved synthesis of graphene oxide. ACS Nano (2010) doi: 10.1021/nn1006368. Copyright (2010) American Chemical Society.

Graphite, the precursor of GO, has a highly ordered crystalline structure composed of layers of sp^2^ hybridized carbon atoms connected within each layer by covalent and metallic bonds and by weak Van der Waals interactions between the layers. As a consequence, graphite is anisotropic, being a good electrical and thermal conductor in-plane and a weak electrical and thermal conductor perpendicular to the plane. The carbon layers in graphite are known as graphene layers (Chung, 2002). Anisotropy allows graphite to undergo chemical reactions called intercalation reactions in which the reagents are inserted between the graphene layers of graphite to form graphite intercalation compounds (GICs) that are electrically more conductive than graphite and then develop into other compounds like graphite oxide. Because of the considerable thermodynamic stability of graphite, the conversion of graphite to graphite oxide requires very drastic reaction conditions involving concentrated acidic media and strong oxidizing agents. The recipes for graphite oxidation still used today, although with some modifications, are almost all based on three main methods: Brodie’s method of 1855 (Brodie, 1858), Staudenmaier’s method of 1989 (Staudenmaier, 1899), and the Hummers method of Hummers and Offeman (1958). Brodie’s method consists of heating graphite at 60°C for 3-4 days in a mixture of potassium chlorate, an oxidizing agent, in fuming nitric acid. The oxidation step must be repeated for 4^_^7 cycles and before each step, the partially oxidized product must be isolated, washed, and dried. Staudenmaier works on Brodie’s method to speed up the reaction and increase yield. He discovered that by replacing fuming nitric acid with a mixture of concentrated sulfuric acid: fuming nitric acid 3:1 and slowly adding potassium chlorate in multiple portions, the reaction continues in a single vessel. However, this reaction requires 4 days to complete. More than 100 years after Brodie’s discovery, Hummers and Offemann proposed a new, faster, and safer method for graphite oxidation. This method involves three reaction steps at controlled temperatures. At low temperature (below 5°C) it occurs the slow addition of an excess of potassium permanganate (three eq.) to a suspension of graphite and sodium nitrite in concentrated sulfuric acid. The reaction continues for 30 min at mid-temperature (∼35°C) and the mixture becomes more homogenous. After this time, warm water is added to the mixture causing heat generation, the temperature reaches 98°C and the reaction is maintained at this temperature for 15 min by an exothermal heat, then the reaction is quenched with a hydrogen peroxide solution to reduce the residual permanganate and manganese dioxide to soluble colorless manganese sulfate. The formation of a bright yellow pasty mixture is evidence of the successful conversion of pristine graphite in GO. The oxidation products obtained with the three methods differ slightly in chemical composition and degree of oxidation. In general, GO obtained with the Hummer method has a higher degree of oxidation as revealed by the lower carbon to oxygen ratio (GO-Hummers 1.12 < GO-Staudenmaier 1.77 < GO-Brodie 2.52) and by the higher spatial distance between the layers observed in the XRD patterns (GO-Hummers 0.8133 nm > GO-Staudenmaier 0.7226 nm > GO-Brodie 0.7084 nm) (Shamaila et al., 2016). Although the methods using KClO_3_ and HNO_3_ suffer from long reaction times and the evolution of acid fog resulting from fuming nitric acid, and the highly explosive ClO_2_ gas generated when chlorate is mixed with strong acids, they remain the most powerful and well-known oxidative methods for producing GO on a preparative scale (Brisebois and Siaj, 2020). Instead, the fastest, easiest, and safest Hummer method is the most widely used approach to obtain graphite oxide on a large scale. Because of its satisfactory characteristics, this method has been widely used to investigate the mechanism of GO formation, as well as widely revisited and modified. One of the most popular modifications of the Hummers method was proposed by Marcano et al. (2010) The Marcano method, known as the improved Hummers method, involves using a quantity of oxidizing agent (KMnO_4_) twice as much as the Hummers method, and the replacement of sodium nitrate with phosphoric acid (H_3_PO_4_). The use of a higher amount of oxidizer results in a product with a higher degree of oxidation, as evidenced by the high ratio of alcohol/epoxide (∼60 ppm) signals and graphitic sp^2^ carbon signal (130 ppm) in Solid-State ^13^C NMR spectra (Figure 2) of GO produced with the improved Hummer method (IGO) compared to NMR spectra of GO produced with Hummer method (HGO) and modified Hummer method (HGO+) with additional KMnO_4_. The use of phosphoric acid, instead, allows obtaining a product with a more regular structure and with a greater number of isolated aromatic rings preserved, as evidenced by the overall absorption in the UV/Vis spectra of the three samples (Figure 2).

Also, the elimination of nitrate from the synthetic procedure avoids the evolution of NO_2_ and N_2_O_4_ toxic gases. Chen et al. compared GO samples obtained from the same graphite source with the Hummer method without or with the use of NaNO_3_, demonstrating that the exclusion of sodium nitrate from the reaction formula does not affect the chemical-physical properties (dispersibility, chemical structures, thicknesses, and lateral dimensions) of the product and the overall yield (Chen et al., 2013a). The authors provide a convenient method for the purification of wastewater collected from the GO purification process. The pollutant Mn^2+^ ions from wastewater can be easily converted into a precipitate of Mn_3_O_4_ by adding KOH. The post-treatment of waste from nanomaterial production processes is a low considered issue, but essential for commercialization. Another version of the modified Hummers method without sodium nitrate consists of pre-oxidization of graphite flakes with a mixture of sulfuric acid, phosphorus pentoxide P_2_O_5_, and persulfate ions (S_2_O_8_^2–^) for 5 h at 80°C (Batalha et al., 2016). The oxidative pre-treatment increases the distance between the graphite layers in the graphite and renders it more available for the subsequent oxidation process. This two-step method results in GO samples with higher oxygen content. The increased interest in the properties of this material encouraged researchers to look for more rapid, cost-effective, green (free of toxic gases and polluting heavy metals), and safe (no risk of explosion) synthesis methods. In this scenario, oxidizers other than potassium permanganate were considered. For example, benzoyl peroxide (BPO) heated to 110°C in an open vessel with graphite powder oxidizes the pristine graphite in 10 min under acid and metal-free conditions (Shen et al., 2009). Although this method is highly efficient, the high instability of benzoyl peroxide and the structural damage in the oxidation product due to the high operating temperature render this procedure not applicable on a large scale. Peng et al. (2015) proposed a completely new method for GO synthesis using potassium ferrate (K_2_FeO_4_) as oxidant. K_2_FeO_4_ has a higher oxidation efficiency than KMnO_4_ in an acid environment, it can be handled without risk of explosion at temperatures as high as 100°C and it does not generate toxic or polluting by-products. In this approach, highly water-soluble GO is obtained after only 1 h stirring of a mixture of concentrated sulfuric acid, K_2_FeO_4_, and graphite flake at room temperature. The GO sheets prepared with K_2_FeO_4_ have a single layer morphology [∼0.9 nm thickness as measured by Atomic Force Microscopy (AFM)] with an average numerical width of ∼10 μm (from Scanning Electron Microscopy (SEM) images) and a degree of oxidation nearly equal to that of the GO produced using KMnO_4_. The authors claim that the high oxidation and exfoliation state is due to the synergy between FeO_4_^2–^ ions and atomic oxygen [O] produced *in situ*. Since the reaction process is extremely simple and requires no energy transfer (neither heating nor cooling), it is straightforward to scale up. Besides, the work-up of this method allows to recover the sulfuric acid used for centrifugation and to reuse it for at least another 10 times without affecting the reaction time and the quality of the product. This eco-friendly, safe, highly efficient, scalable, and low-cost approach is unfortunately difficult to reproduce because the strong oxidizing power of ferrate (VI) is directly related to its instability, particularly in acidic environments (Sofer et al., 2016). In high-acid aqueous solutions, potassium ferrate (VI) decomposes in a few seconds via an autocatalytic process, which limits its applications in chemical synthesis. Moreover, it is not widely commercially available and its synthesis involves the use of chlorine gas which is highly toxic. Among the modern approaches, particular attention is focused on the production of GO by electrochemical exfoliation which is simple, environmentally friendly, and substrate-free. Typically, GO flakes are generated taking advantage of the electrical conductivity of graphite (e.g., foil, rod or flakes, flexible paper) in aqueous electrolytes (H_2_SO_4_ or H_3_PO_4_). A typical apparatus for electrochemical exfoliation of graphite consists of an electrochemical cell with two electrodes of graphite in an aqueous solution of electrolytes (Liu J. et al., 2013). At first, a static potential of 1 V is applied to the two electrodes to wet the electrode surface and facilitate the accumulation of charges around the anode. The applied potential is then increased to +7 V and maintained for 5-10 min. At this potential value, the anions in the electrolytic solution are guided to intercalate between the galleries of the graphene layers. By alternating the potential between +7 and -7 V it is possible to obtain exfoliation of both electrodes. After only a few minutes of electrolysis, a change in color of the aqueous electrolyte from transparent to dark is observed, which indicates the formation of GO. Similar to chemical oxidation, various sources of graphite or metals such as platinum and titanium can be used as electrodes and a variety of electrolytes including inorganic aqueous solutions, surfactants, molten salts, and ionic liquids. The electrochemical exfoliation of graphite is a tunable process because the chemical and chemical-physical properties of the oxidation product (morphology and degree of oxidation) can be easily adapted by adjusting the experimental set-up (type and geometry of the electrodes, solution, electrolytic, applied voltage, time and temperature). An extensive review of electrochemical methods for GO synthesis has been provided very recently by Fang et al. (2019).

## Mechanism of Graphene Oxide Formation and Breakage

**FIGURE 3:**
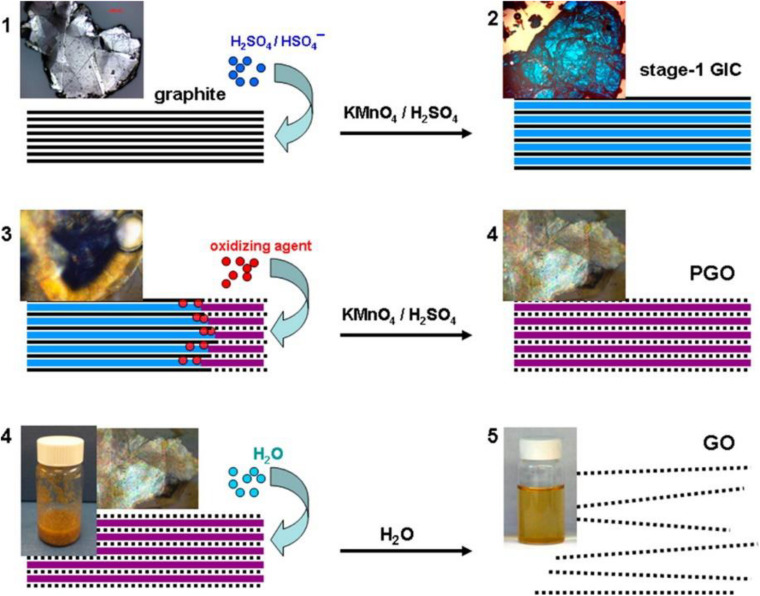
Stage of conversion of bulk graphite in GO solution with corresponding micrographic images. (1) Graphite intercalation compound formation; (2) graphite intercalation compound conversion in pristine graphite oxide; (3) pristine graphite oxide conversion to GO. The solid black lines represent graphene layers; dotted black lines represent single layers of GO; wide blue lines represent H_2_SO_4_/HSO_4_^–^ intercalant; wide purple lines represent a layer of the mixture of H_2_SO_4_/HSO_4_^–^ intercalant with the reduced form of the oxidizing agent. Reproduced with permission from Dimiev, A. M. & Tour, J. M. Mechanism of graphene oxide formation. ACS Nano (2014) doi: 10.1021/nn500606a. Copyright (2014) American Chemical Society.

Knowing the formation mechanism of GO is a key step in the development of new materials with the desired properties. However, despite the remarkable progress in understanding the chemistry and structure of GO, the mechanism of its formation received little attention from the scientific community. The majority of the studies reported are theoretical and focus on the introduction of oxygen atoms into the graphene lattice with the formation of C-O bonds (Gao et al., 2011; Li C. et al., 2020). In an elegant work of Dimiev and Tour (2014), Dimiev and Tour have illuminated the steps that occur during the oxidation of graphite with potassium permanganate into concentrated sulfuric acid both within the solid graphite (between the graphene layers) and at the solid/liquid interface. Starting from the assumption that the characterization of the final graphite oxide obtained with the Hummer method, washed with water and dried is not very informative in mechanistic terms, the intermediate products that are formed in the various step of additions of permanganate have been isolated. The characterization, through optical microscopy and Raman spectrometry, of the obtained intermediates has permitted to identify of three distinct independent steps in the chemical oxidation of graphite with strong oxidants in concentrated mineral acids. The first step is the classic graphite intercalation reaction in which H_2_SO_4_ molecules and HSO_4_^–^ ions intercalate between graphite galleries without creating an orderly structure. The intercalation compound is formed after a few minutes that the graphite is exposed to the acid medium and imparts an intense blue color to the graphite. The intercalation is a necessary step for successful oxidation because it increases the distance between the graphene layers in the graphite making the tunnels between the layers accessible to the oxidizing agent. The second step is the conversion of GIC into the oxidized form of graphite called pristine GO. In this step, the oxidizing agent molecules are inserted into the pre-occupied graphite galleries. Optical microscopies of the graphite flakes isolated at this stage clearly show that the conversion from intercalation compound (blue color at the center of the flake) to pristine GO (pearly white color at the edges) propagates from the edges of the flakes to the center (Figure 3). Theoretically, in the conversion from the intercalation product to the oxidation product, the oxidant molecules should replace or intercalate with the acid molecules present in the graphite interlayers. The experimental data show, instead, that the speed of the oxidation reaction is greater than the rate of diffusion, in other words, before the oxidizing agent diffuses between the graphite layers, it reacts rapidly with the nearby carbon atoms. So the pristine graphite oxide formation is the step that determines the rate of the entire GO formation process.

**FIGURE 4:**
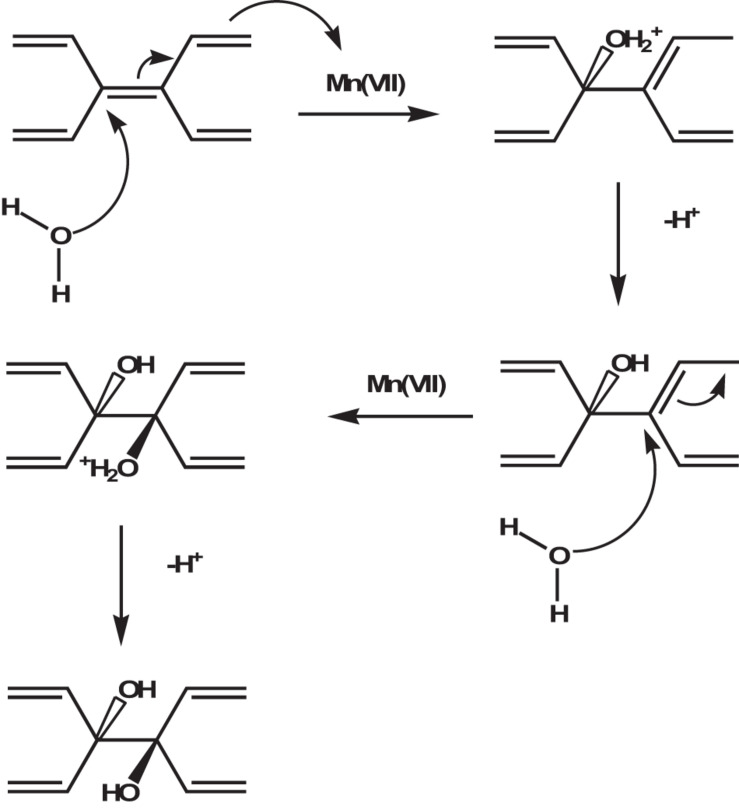
Role of water in the oxygenation of graphite during the synthesis of GO. The withdraw of electrons by Mn (VII) species and the nucleophilic attack by H_2_O on the as-formed positively charged carbon atoms occur simultaneously as a concerted process. Adapted from Dimiev, A. M., Shukhina, K. & Khannanov, A. Mechanism of the graphene oxide formation. The role of water, “reversibility” of the oxidation, and mobility of the C–O bonds. Carbon N. Y. (2020) doi: 10.1016/j.carbon.2020.05.005.

The final step is the conversion of pristine graphite oxide to GO by reaction with water during quenching and washing procedures. At this stage the graphite oxide, if sufficiently oxidized, delaminates spontaneously into single atomic layer sheets. To obtain exfoliation hydrogen bonds and electrostatic interactions between water and GO must overcome the electrostatic interaction between graphene layers and intercalants (within each layer) and water molecules must be able to hydrolyze covalent sulfates that cross-link neighboring layers. Very recently the same authors have corrected the mechanism by reconsidering the role of water in the formation of GO. To investigate the nature of the effective oxidizing species attacking graphite layers, they found that the rate of oxidation reaction increases by a factor of 12 when the reaction is carried out in slightly diluted sulfuric acid (92%-88%) rather than in commercially available H_2_SO_4_ at a concentration of 95%-98% (Dimiev et al., 2020). This observation opens an important perspective on the reaction mechanism. Following the same procedure of isolation and characterization of intermediates, the authors conclude that the species attacking the carbon atoms in the Hummers method are water molecules and not oxidant molecules. However, manganese species (VII) cannot be completely omitted from the reaction equation because they are consumed during the reaction and because once the first equivalent of permanganate is consumed, the reaction does not go on unless another one is added. The authors claim that the reaction occurs directly between the H_2_SO_4_-graphite intercalation compound and water according to a mechanism very similar to the hydration of aromatic hydrocarbons in acid media (Figure 4) where water nucleophilically attacks the carbon atoms of graphite and the Mn (VII) species accept the electrons that are released, reducing to Mn (IV).

**FIGURE 5:**
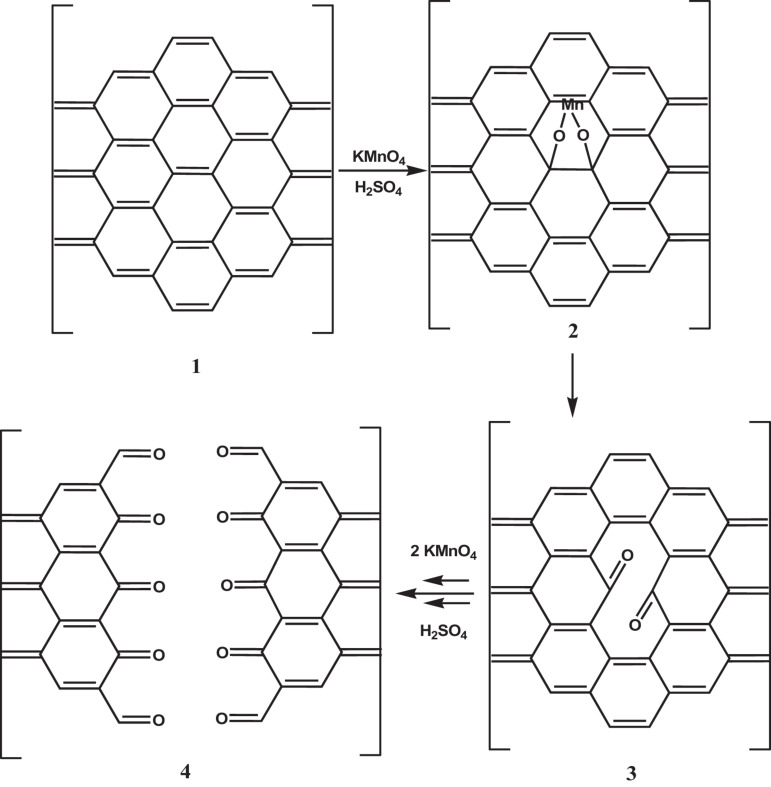
Mechanism of the GO breakage by increasing the amount of oxidants or prolonging the reaction time. A rate-determining step is the manganite ester 2 formation, which oxidizes further to afford dione 3 in the dehydrating medium. The juxtaposition of the buttressing ketones distorts the β,γ-alkenes, making them more prone to the next attack by permanganate. As the amount of oxidants or the oxidation time increases, the bond-angle strain induced by the enlarging hole increase, and finally, it results in the nanotube opening 4. Adapted from Kosynkin, D. V. et al. Longitudinal unzipping of carbon nanotubes to form graphene nanoribbons. Nature (2009) doi: 10.1038/nature07872.

Since the formation of GO is a process controlled by the rate at which oxidizing molecules or water molecules diffuse between the graphite layers, smaller graphite flakes oxidize faster than larger ones. In addition, since no any graphite flake is equal to another, it is not surprising that GO is a material with a wide size distribution ranging from a few nanometers to hundreds of micrometers. However, it is possible to select GO sheets of appropriate size through post-synthesis methods or reduce the sheet size by adjusting the parameters of the chemical reaction or by using mechanical promoters. The structural characteristics of the obtained nGO and the breaking mechanism are very dependent on the method used for breaking. In general, when acting on the chemical reaction (by varying the amount of oxidants, the time or temperature of the reaction) GO nanosheets with a higher oxygen content are obtained (Zhang et al., 2013), while when using physical methods to promote breakage (ultrasonication) nanosheet with a higher C/O ratio is obtained (Gonçalves et al., 2014). These experimental observations reflect a different breaking mechanism. As it is known, crystalline and amorphous regions coexist in the structure of GO. The breakage of GO into sheets with lower lateral size occurs through the formation and propagation of cracks over defects zones, in particular regions with the sp^3^ bond like -C-OH and O-C-O. When GO is fragmented by increasing the amount of oxidants or prolonging the reaction time, the reaction mechanism is similar to that of oxidizing olefins with KMnO_4_ in an aqueous solution and has been demonstrated experimentally following the gradual opening of carbon nanotubes as the exposure time of the system in the oxidizing medium increases (Kosynkin et al., 2009). This mechanism involves the formation of a manganate ester in the rate-determining stage of the reaction. The ester further oxidizes to dione in the dehydrating medium and distorts the nearby double bonds making them more available to the next permanganate attack. The ketones can be further converted, through their O-protonated forms, to the carboxylic acids that will line the edges of the nanosheet (Figure 5).

**FIGURE 6:**
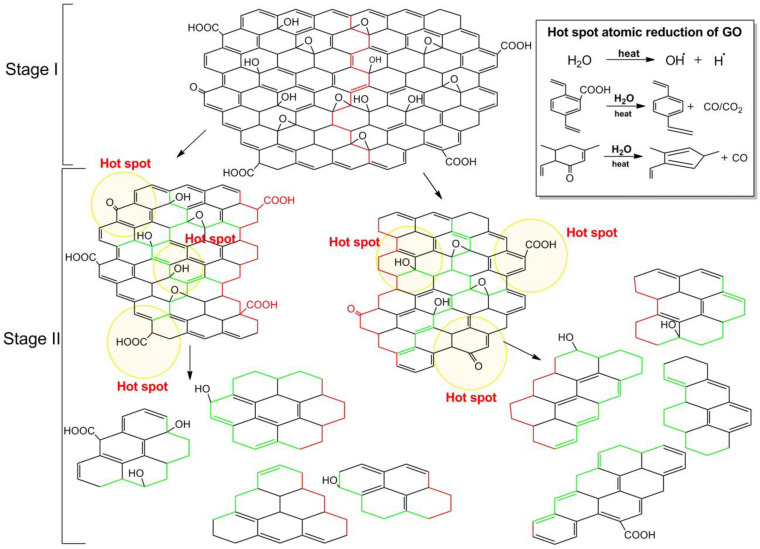
Mechanism of the breakage by ultrasonication. The first stage illustrates the chemical bond breaking from the defective zone (red line), the second stage illustrates the further dimensional and degree of oxidation reduction as ultrasonication increases. Inset shows the hot spot atomic reduction reactions of GO. Reproduced from Gonçalves, G. et al. Breakdown into nanoscale of graphene oxide: Confined hot spot atomic reduction and fragmentation. Sci. Rep. (2014) doi: 10.1038/srep06735.

When the breaking of GO occurs by applying an external energy source, the mechanism involves the breaking of chemical bonds. The breaking starts from defects zones, in particular regions with the sp^3^ bonds like -C-OH and O-C-O, as previously said. After propagation of cracks, smaller sheets are obtained (Figure 6). If the process continues a smaller, but also more hydrophobic material will form. The use of ultrasound generates a local increase in temperature and pressure that promotes the decomposition of water molecules in the medium into hydroxyl radicals. ⋅OH radicals radicals have the potential to reduce carboxylic and carbonyl groups by restructuring the aromatic carbon network and forming a more hydrophobic material (Gonçalves et al., 2014). This mechanism is defined as confined hot spot atomic reduction of GO.

## Strategies for Size-Control of Graphene Oxide Sheets

The main methods for controlling the size of nGO sheets are summarized in Table 1. Generally, they can be categorized as direct controllable synthesis and post-synthesis separation.

**TABLE 1:** List of methods for controlling the size of nGO sheets.

	**General Method**	**Specific strategy**	**Feature size**	**References**
Direct Controllable Synthesis	Different Precursors	**Graphite Nanofibers:** very small graphite plates (diameter about 130 nm and length up 5 μm) stacked in particular conformation	100-50 nm	Luo et al., 2010
		**Arc-discarge Material:** carbon material highly flexible and defective obtained by electrical breakdown	20-40 nm	Rosillo-Lopez and Salzmann, 2016
		**Aphanatic Graphite:** kind of graphite with disorder structure obtained by thermal decomposition of deep metamorphic	4.5 nm	Shen et al., 2020
	Reaction Parameters	**Time and Oxidant Amounts:** increase in the amount of oxidizing agent and timescale of oxidation in the same batch	50-20 nm	Zhang L. et al., 2009, Zhang et al., 2013
		**More Oxidation Cycles:** for each cycle the obtained product was isolated, purified and re-oxidized	10-5 nm	Fan et al., 2015
	Physical Promoters	**Ultra Sonication:** application of sound energy in a water bath or through probes to break GO sheets in low temperature conditions	100-500 nm	Farazas et al., 2018; Méndez-Romero et al., 2020
		**Ball Milling:** graphite with or without solvents or oxidants was mechano-chemical oxidized in a rotating cylindrical jar filled with loose grinding balls.	depending on conditions from μm to 30-50 nm	Posudievsky et al., 2012; Mahmoud et al., 2018; Luo D. et al., 2019
Post-synthesis Separation	Centrifugation	**Differential Centrifugation Method:** GO sheets to be separated was divided in several fractions according to their size by varying the centrifugal force applied	250-900 nm	Chen et al., 2013b; Huang and Yuan, 2016
		**Density Gradient Centrifugation:** GO to be separated was placed on the surface of a vertical column of liquid with increased density from top to bottom and centrifuged. The particles migrated through the solvent gradient and settled where their buoyancy density equaled that of the gradient	40-500 nm	Sun et al., 2010; Li et al., 2013; Bidram et al., 2016
	Flocculation by Solvent	**Protonation in Organic Solvents:** large GO sheets precipitated selectively in organic solvents by protonation of carboxyl groups on the edges	500-100 μm	Zhang et al., 2015
		**pH Assisted Precipitation:** nGO sheets were recovered from the strongly acidic precipitate obtained from the work-up of the classic oxidation reaction by increasing pH value to 1.7 with 1M NaOH	90 nm	Hu et al., 2015

### Direct Controllable Synthesis

#### Different Precursors

**FIGURE 7:**
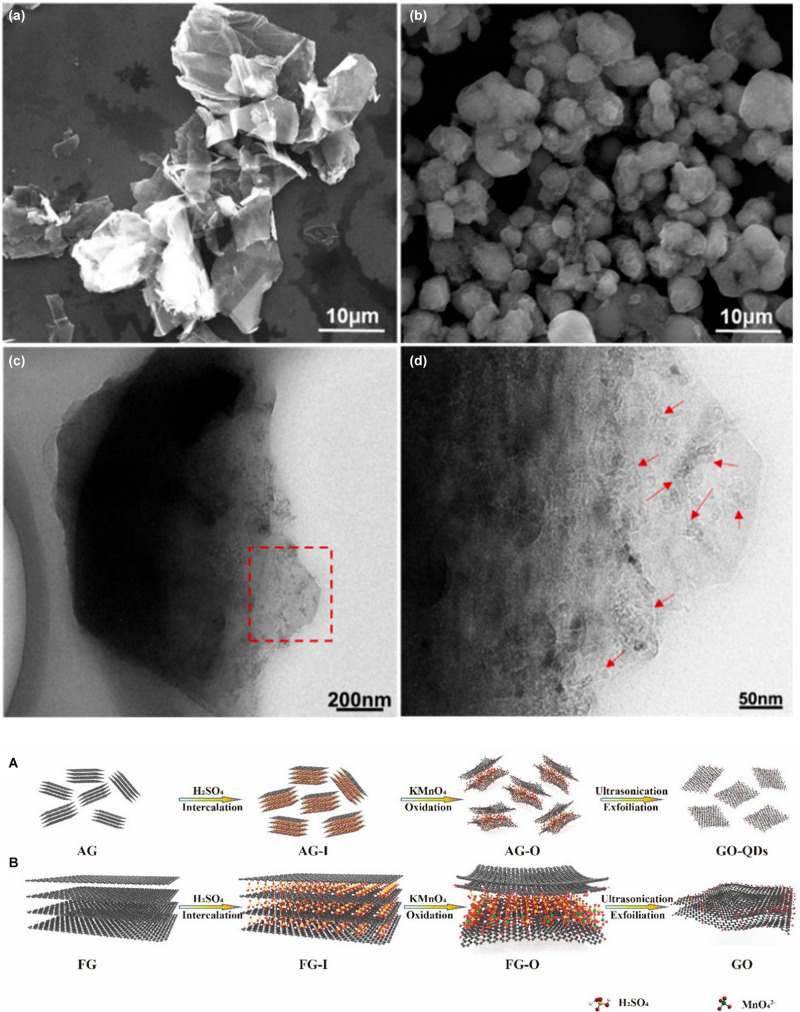
**(a)** SEM image of natural graphite, **(b–d)** SEM and TEM images of aphanatic graphite, the red arrows indicate the small graphite nanocrystal. Schematic comparison between the exfoliation mechanism of aphanatic graphite **(A)** and natural graphite **(B)** with the same amount of intercalator and oxidizer. Adapted from Shen, S. et al. Graphene quantum dots with high yield and high quality synthesized from low-cost precursor of aphanitic graphite. Nanomaterials (2020) doi: 10.3390/nano10020375.

As source of GO production, graphite is intercalated with the acid medium, converted into its oxidized form by oxidants, and finally exfoliated by reaction with water. Graphite can be divided into natural graphite, organic matter rich in carbon, and artificial graphite, a synthetic matter obtained by heating of petroleum coke, coal-tar pitch, or oil. The particles are polycrystalline in both cases and composed of various single-crystalline domains (depending on the size of the domains, graphite is distinguished in micro- and macro-crystalline graphite). However, these domains are typically oriented in the same direction in natural graphite and randomly oriented in synthetic graphite (Asenbauer et al., 2020). The raw material characteristics play an important role in the properties of the oxidation material. For instance, natural graphite has a more orderly, but also wider crystalline structure, which facilitates the intercalation of oxidants between the graphene layers and the GO from natural graphite has a yield of exfoliation in water that is almost twice as high as the GO from artificial graphite (Botas et al., 2013). The size of graphite precursors, on the other hand, affects both the efficiency of the oxidation process and the lateral dimensions of GO sheets. McAllister et al. (2007) discovered that graphite flakes with a size of around 45 μm are completely intercalated and rapidly oxidized, while larger flakes (∼400 μm) do not intercalate completely and require more time to oxidize. Chen et al. (2016) fractionated graphite powders in three portions at increasing-size using standard sieves and oxidized them with an optimized Hummers method in which the volume of sulfuric acid used was increased as the size of the powders increased. The GO sheets obtained by this process are mostly single-layer and have an extremely narrow size distribution that is highly dependent on the size of their graphite precursors (Chen et al., 2016). Therefore, the selection of the starting material is an important factor in predicting the size of the product obtained after oxidation. Concerning the initial size of graphite flakes used for synthesis size reduction control can be obtained by the same chemical exfoliation method, but with different precursors. Graphite nanofibers are materials produced by the decomposition at temperatures between 450 and 750°C of gases containing carbon and their mixtures on appropriate metal or metal alloy surfaces. These structures consist of very small graphite plates stacked in a particular conformation (parallel, perpendicular, or cornered to the fiber axis) and are widely used in hydrogen storage applications (Chambers et al., 1998). The controlled oxidation of graphite nanofibers yields GO nanosheets with lateral dimensions below 100 nm and high colloidal stability. The graphite nanofibers used in this work have a highly crystalline structure in which the graphene sheets are coin-stacked along the <001> fiber growth direction. The average diameter of nanofibers is about 130 nm, and the length is up to a few micrometers (Luo et al., 2010). In a typical experiment, the nanofibers are first subjected to a pre-oxidative treatment with K_2_S_2_O_8_ and P_2_O_5_ in concentrated sulfuric acid for 4.5 h at 80°C, then naturally cooled, washed with water, and filtered. After pre-oxidation, the graphite nanofibers are oxidized using the modified Hummers method and then purified using an acid: acetone washing procedure. After 2 h of oxidation at 35°C, uniform GO nanosheets with average lateral dimensions around 50 nm are obtained. Increasing the reaction time decreases the average lateral dimensions up to 20 nm obtained in 12 h of reaction. The obtained GO nanosheets have spectroscopic characteristics and chemical properties very similar to micrometric GO sheets but have very different properties in solution, such as surface activity and colloidal stability. Due to the higher charge density caused by their higher edge-to-area ratios, aqueous GO nano colloids are significantly more stable. A colloidal dispersion of nGO (1 mg mL^–1^) remains stable even after centrifugation at 10000 rpm for 30 min, while a colloidal dispersion of micrometric GO at the same concentration begins to precipitate at 5000 rpm. Although the properties of the product obtained are very interesting, the massive pre-oxidation step and the long oxidation times do not allow estimating the actual role of the precursor in achieving dimensional control. Rosillo-Lopez and Salzmann proposed a simple and gentle chemical oxidation route to obtain high purity nGO that involves no sulfuric acid and potassium permanganate and requires no long purification steps (Rosillo-Lopez and Salzmann, 2016). Their synthetic methodology involves the use of Arc-discharge (ADC) material consisting of single-wall carbon nanotubes (SWCNT), multi-wall carbon nanotubes (MWCNT), and graphitic carbon obtained through the arc-discharge technique which consists in the application of a direct current between two graphite electrodes placed in an atmosphere of inert gas. Due to the high temperature between the electrodes the graphite at the anode sublimates and at the cathode a dark deposit containing the ADC material is formed (Rakhi, 2018). The carbon nanostructures obtained with this technique are highly flexible and have many small defects. The material is simply refluxed in a 1:1 solution of distilled water: nitric acid 6M for 20 h, then diluted with water and filtered. Neutralizing the dark brown filtrate obtained by carefully adding NaOH pellets, the precipitation of sodium-containing nGO is observed. The solid is vacuum filtered, purified by dialysis, and freeze-dried to obtain a dark brown nGO solid with a 21% yield. AFM and transmission electron microscopy (TEM) images show that the obtained flakes have a double-triple-layer morphology with an average lateral size between 20 and 40 nm. The XPS survey spectra of GO flakes show that the final material contains only carbon and oxygen and the nitrogen impurities coming from the method are completely absent. The high purity of the obtained product combined with the simple procedure and the absence of harmful by-products makes this procedure very competitive, even if the yield is low and, during the reaction, there is the development of nitrogen oxide fumes (NO_*x*_). The authors, through thermal annealing investigations of nan-GO under high vacuum, also hypothesized the formation of a cyclic carboxylic anhydride during the thermal annealing of their GO. The anhydrides of the carboxylic acids are highly reactive chemical species that may constitute sites for the subsequent chemical functionalization with purpose-specific nucleophiles. Very recently graphene oxide quantum dots with an average lateral size of about 4.5 nm and an average thickness of ∼ 3 nm have been successfully synthesized using the Hummers method with sodium nitrite starting from aphanatic graphite (Shen et al., 2020). Aphanatic graphite is a kind of graphite ore that is composed of carbonaceous material by thermal decomposition of deep metamorphic products (such as from coal deterioration). It has lower thermal conductivity, lubrication, and oxidation resistance than fully crystalline graphite and therefore is much lower in price than flake graphite. Furthermore, unlike graphite flakes which have a layered structure with a length of each layer of about 35 μm, the aphanatic graphite has a particle structure with an average particle diameter of about 5 μm. Within each graphite aphanatic particle, there are small graphite nanocrystals not completely exfoliated during the formation period with dimensions less than 10 nm (Figure 7A). These defective characteristics allow the synthesis of graphene quantum dots by oxidation.

As explained above, in fact, the oxidation process of graphite under the conditions of the Hummers method is diffusion-controlled. With the same oxidant, the smaller and less crystalline graphite flakes are oxidized faster and more homogeneously than the large and highly crystalline flakes because the resistance to diffusion between the layers is lower (Figure 7B).

#### Reaction Parameters

Graphene oxide is obtained by the oxidation of graphite flakes in strong mineral acids with potent oxidizing agents. As pointed out above the degree of oxidation, i.e., the O/C ratio, affects structural properties such as the degree of exfoliation, the content of defects, the number of functional groups, and also the size of the sheets. More precisely, the degree of oxidation is linearly correlated with the size of the flakes, therefore, increase the kinetics of oxidation by changing the reaction parameters of the classical method (stoichiometric amount of reagents, temperature, and reaction time), it is achievable GO flakes with smaller lateral dimensions than the starting graphite. The effect of the oxidation time and the amount of oxidants in the dimensional control of GO sheets obtained with the Hummer method was first studied in 2009. In this experiment, the graphite flakes are oxidized using the Hummer method with KMnO_4_ and NaNO_3_ in H_2_SO_4_. In the first reaction step, 4.5 eq. of KMnO_4_ are added to the mixture of graphite, sulfuric acid, and sodium nitrate, and the reaction is continued for 5 days at room temperature. A further 2.25 eq. of KMnO_4_ are then added to the resulting mixture and stirring is continued at room temperature for 5 days. The step of adding the 2.25 eq. of oxidizer and stirring for 5 days is repeated twice more and, at each step, the reaction products are isolated, purified, and characterized (Zhang L. et al., 2009). The single-layer GO sheets obtained with this extensive procedure exhibit a regular decrease in average lateral size as oxidants and reaction time increase. Using a similar procedure, Zhang et al. (2013) prepared GO nanosheets with a lateral size of less than 50 nm after three cycles of oxidation with KMnO_4_ of pre-oxidized graphite with P_2_O_5_ and K_2_S_2_O_8_. The TEM images of GO nanosheets obtained after three oxidation cycles show single layer sheets with rough edges and uniform lateral dimensions below 50 nm. The colloidal solutions of the nanosheets are stable in a wide range of pH values from 10 to 4, as evidenced by the value of their zeta potential which remains always below -30 mV. GO colloids are only stable when the zeta potential was below -30 mV. The colloidal stability in aqueous solution at different pH values is due to the higher degree of oxidation. FT-IR and XPS analyses indicate a higher content of oxygenated groups compared to GO samples obtained with lower oxidation steps. Moreover, GO nanosheets obtained after three oxidation steps emit a strong fluorescence at 520 nm when excited at 400 nm, which is almost six times stronger than nanosheets obtained after one or two oxidation cycles at the same concentration. The nanometer size, colloidal stability, and fluorescence emission are the characteristics required for biological applications of GO. The authors have also analyzed the cellular uptake and cytotoxicity of these materials on cells, finding that these events are similarly size-dependent. It is interesting to note that Fan et al. (2015) report the synthesis of photo-luminescent graphene oxide quantum dots of circular shape and diameter less than 10 nm by processing the purified GO obtained with the modified Hummer method to a further oxidation procedure with the same method. Tuning the reaction parameters is not a cost-effective solution for large-scale GO synthesis because it is time-consuming and it requires a large excess of acids and oxidizing agents.

#### Physical Promoters

##### Ultrasonication

**FIGURE 8:**
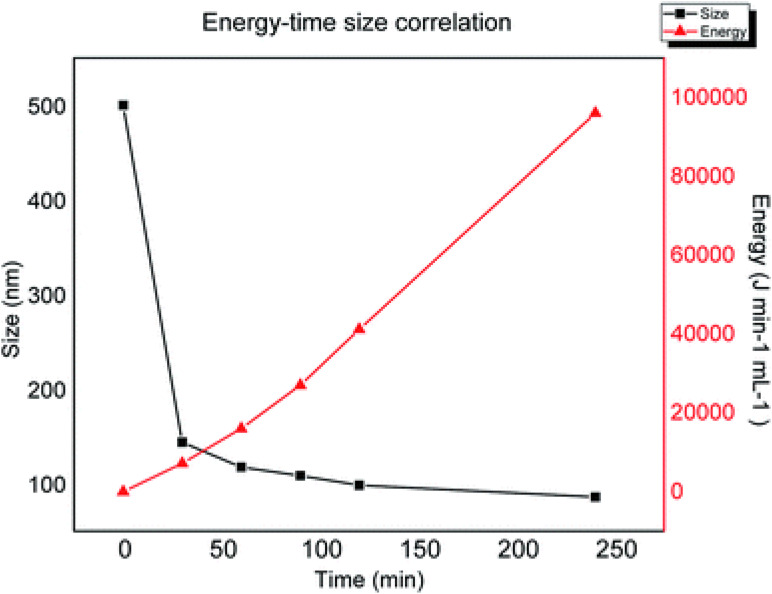
Decrease in the lateral size of graphite flakes versus energy applied and sonication time. Reproduced from Méndez-Romero, U. A., Pérez-García, S. A., Fan, Q., Wang, E. & Licea-Jiménez, L. Lateral size reduction of graphene oxide preserving its electronic properties and chemical functionality. RSC Adv. (2020) doi: 10.1039/d0ra04726k.

The application of ultrasound and mechanical shaking are the most commonly reported procedures to perform the exfoliation of pristine graphite oxide in water in the final step of chemical oxidation. Sonication is only necessary for GO samples that are not fully oxidized, made from graphite samples with large particle size, but if the as-prepared GO is sufficiently oxidized it should spontaneously delaminate into single-layer sheets by simple stirring in water (Dimiev and Eigler, 2016). However, the sonication of aqueous GO dispersions is an efficient method to control the size of the flakes. Sonication is a process in which sound energy is applied to agitate particles in a medium. It is usually performed in a water bath in which samples are placed or through probes immersed directly in the sample to be sonicated. During sonication, vacuum bubbles are formed in the liquid. When the vacuum bubbles reach a certain size they collapse violently creating a high-pressure acoustic wave. The implosion of the bubble results in shear forces from cavitation and stress waves, extreme temperature and pressure, fast cooling times, and high-speed liquid jets (Han et al., 2014). These intense local forces break the GO sheets according to the mechanism described above. Qi et al. (2014) reported a facile sonochemical method for the preparation of size-specified GO sheets started from large GO sheets with a wide dimensional distribution from a few hundred nanometers up to about 5000 μm, obtained via a mild Hummers method performed at low temperature (35°C) for two h. The obtained GO flakes are dispersed in water at a concentration of 0.5 mg/mL and subjected to bath ultrasonication for a selected time (3, 6, 9 h). The temperature of the water in the ultrasonic bath must be kept below 35°C to avoid the reduction of GO (Qi et al., 2014). The lateral size of the resulting GO samples shows a Gaussian distribution with the maximum decreasing regularly with increasing ultrasound time. Moreover, the SEM images of the samples show that at the early stage of ultrasonication, the sheet size decrease sharply and then the decrease slows down with the increase of ultrasonication time. Another interesting phenomenon observed during the ultrasonic process is that the color of the solution becomes more intense as the ultrasonic process increases, indicating that this process does not affect only the lateral dimension of the sheets but also the chemistry. An accurate characterization of the obtained materials has allowed establishing, for the first time, that ultrasounds are effective in exfoliating GO sheets, but also in increasing the degree of oxidation of samples not completely oxidized through mechanical shear forces created by the collapse of cavitation bubbles. The effect of sonication in reducing the lateral size of GO sheets has also been demonstrated with the GO obtained with the Marcano method (Farazas et al., 2018). As we have stressed previously when considering the reduction of GO size by ultrasonication, the possible chemical reduction must be taken into account. The product that is obtained is a reduced graphene oxide, rGO, in which sp^2^ domains restored by reduction and residual oxygenated functional groups coexist. Mèndez-Romero et al. have recently reported a simple but highly effective approach to prepare GO around 100 nm in lateral dimension and high concentration by ultrasound in low-temperature conditions, without alteration of electronic properties and excellent solubility in water (Méndez-Romero et al., 2020). Their procedure consists of sonicating an aqueous dispersion of GO (3mg mL^–1^) with an ultrasonic probe operating at a 40% amplitude while keeping the temperature strictly controlled at 18°C for 4 h. The dynamic light scattering (DLS) analysis of the samples taken at regular time intervals (every 30 min for a total of 4 h) shows that the lateral size of the flakes decreases significantly in the first 2 h of the process from 500 nm to about 100 nm, reached 100 nm, the size decreases more slowly as the time of sonication increases. As described above, when the reduction of the lateral dimensions of the GO is achieved by applying an external energy source, the breaking mechanism begins at the defective sites (sp^3^ hybridized carbon atoms) and then proceeds through the formation of hot spots that result in a further decrease in size, but also in the degree of oxidation. If, however, the temperature of the experiment is carefully controlled, it is possible stopping the reduction in size at an intermediate step and avoiding a decrease in the O/C ratio. The plot in Figure 8 shows the progression in size reduction depending on the energy during the time of the experiment. At the initial stage, a small energy increase induced strong decrease in a short time because there are many defective sites. At the plateau, the defective sites are now depleted and the further increase in energy does not cause a strong decrease in size because the controlled temperature does not allow the formation of hot spots.

Raman and XPS analyses allowed establishing that the sonication at a low temperature preserves the C/O ratio and consequently the chemical stability, the bandgap, the electronic energy levels, and the functionality. Reducing the lateral dimensions of GO by application of acoustic waves is, therefore, an effective approach, but must be used carefully to avoid structural damage to the product caused by the reduction due to local heat generation or by the inclusion of impurities released by the deterioration of immersion probes under operating conditions.

##### Ball mill

**FIGURE 9:**
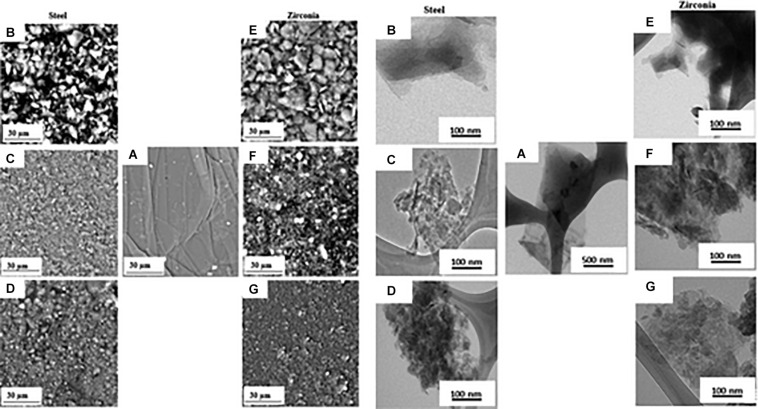
SEM micrographs (left) and TEM images (right) of pristine graphite **(A)**, GO milled with stainless steel for 6h **(B)**, 16h **(C)**, 24h **(D)**, GO milled with zirconia steel for 6h **(E)**, 16h **(F)** and 24h **(G)**. Reproduced with permission from Mahmoud, A. E. D., Stolle, A. & Stelter, M. Sustainable Synthesis of High-Surface-Area Graphite Oxide via Dry Ball Milling. ACS Sustain. Chem. Eng. (2018) doi: 10.1021/acssuschemeng.8b00147. Copyright (2018) American Chemical Society.

The reduction of particle size using a mechanical force is termed mechanical milling (Ullah et al., 2014). The method was developed in 1970 by John Benjamin to synthesize oxide dispersion strengthened capable of withstanding high temperature and pressure (Benjamin, 1970). The process consists of inserting the powder to be ground, eventually together with solvents or surfactants, into a rotating cylindrical grinding jar (ball mill) filled with loose grinding balls (Piras et al., 2019). The rotation of the balls in the jar creates impact forces and shear forces that act in combination to reduce the size of the powder. The mechanical stress produced in a ball mill can therefore be used as a pre-treatment of graphite flakes. Luo D. et al. (2019) prepared GO from large micron-size graphite flake subjected to ball milling. The shiny silver flakes become a fine black powder after ball milling and are quickly dispersed in a lower amount of sulfuric acid than commonly used in the Hummers method indicating the formation of a graphite intercalation compound. The fine powder is more active, and the defects generated by the ball milling process also serve as weak points for intercalation. The oxidation reaction is then conducted by adding the oxidizing agent and keeping the temperature below 5°C by adding dry ice pellets directly into the reaction vessel. The authors declare that the addition of dry ice is the key passage of their method because it can provide enough cooling to avoid the decomposition of the Mn_2_O_7_ surface, the explosive compound formed by the reaction of KMnO_4_ in H_2_SO_4_. After quenching, hydrolysis, and purification, a dark yellow GO solution is obtained which disperses easily in water by ultrasonication and which has the usual spectroscopic features of GO obtained by conventional methods. The majority of GO sheets, observed with electron microscopy, have a single layer with a narrow dimensional distribution around 300 nm. However, comminution using ball mills not only reduces the particle size but the high rotation speed and the collisions of the jars and balls also provide sufficient kinetic energy for the breakdown of the bonds in the aromatic graphite structure. This process introduces functional groups on the edges, surfaces, and basal planes of graphene materials during the milling process (Bharath et al., 2015). Therefore, mechanochemical ball milling can be employed for the production of GO without the need for additional oxidants other than air. Mahmoud et al. (2018) developed a one-step, dry production route to synthesize GO. The authors utilize graphite flakes of 300 mesh and evaluate the effect of two different milling materials (steel and zirconia) and the milling time (6 h, 16 h, and 24 h) on the characteristics of the GO produced (Mahmoud et al., 2018). SEM and TEM micrographs display a change in sample morphology after ball milling. The samples exhibit irregular and stacked layered structure with a reduction in lateral dimensions that increases with increasing milling time, independently of the milling material (Figure 9).

Thermal and Raman analyses show that the GO produced by ball milling has a structure with fewer defects than the GO synthesized by chemical route without contamination of other chemical elements. However, the degree of oxidation obtained with this technique is lower than that of GO obtained by chemical exfoliation of graphite, as evidenced by the red-shift of the absorbance peak in UV-vis spectra. The C/O ratio in GO samples obtained with ball mill decreases with increasing milling time as shown by the XPS spectra but remains lower than the chemically obtained GO samples. This trend is independent of the material used for the jar and milling balls, although the rate of C/O decrease in the material as the milling time increases is more pronounced for stainless steel ball (due to their higher density) than for zirconia materials. One opportunity to increase the degree of oxidation of the GO obtained with this technique could be to mill the graphite together with a chemical oxidant. Dry mechanochemical oxidation of graphite with various solid oxidizers (KMnO_4_, (NH_4_)_2_S_2_O_8_) was reported by Posudievsky et al. (2012) obtaining a degree of oxidation comparable to that of dry methods in which air is used as an oxidizer. In this approach potassium permanganate, in the absence of acid, cannot form an oxidant as strong as manganese anhydride as it is used in the case of graphite oxidation with the Hummers method. Subsequently, the same authors conducted a comparative study of graphite oxidation by potassium permanganate in the absence and the presence of a small, stoichiometrically necessary, amount of sulfuric acid (87%) under identical conditions of the mechanochemical treatment (Posudievsky et al., 2013). The mechano-chemical treatment of the mixture of graphite and the oxidizing solid KMnO4 in the presence of a small amount of sulfuric acid allows preparing the graphite oxide with a high degree of oxidation and with lateral dimensions between 30 and 50 nm. The obtained GO nanosheets have an exceptional dispersibility in aqueous solution up to the concentration of 4.6 mg mL^–1^ without the use of sonication.

### Post-synthesis Separation

#### Centrifugation

**FIGURE 10:**
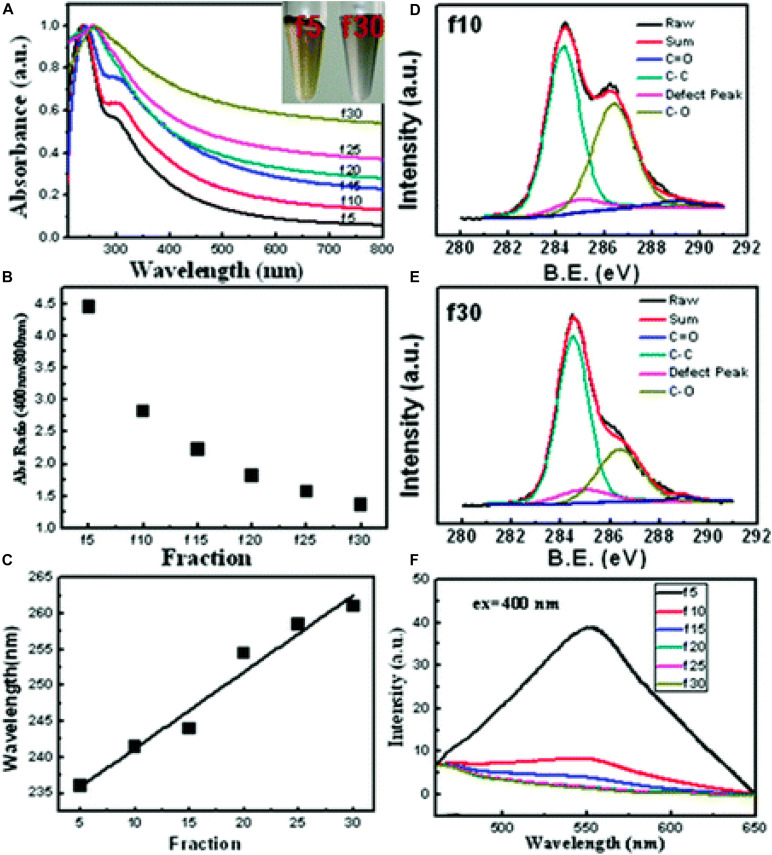
**(A)** UV-vis absorption spectra of GO in different fractions from the density gradient ultracentrifugal rate separation in 20%-66% (w/v) sucrose gradient for 5 min. Inset: photograph of GO (f5 (left) and f30 (right)) after dilution. **(B)** Ratio of absorption at 400 nm to that at 800 nm of GO in different fractions from density gradient ultracentrifugal rate separation in 20%-66% (w/v) sucrose gradient for 5 min. **(C)** Variation in the wavelength of the absorption maximum of GO in different fractions from density gradient ultracentrifugal rate separation in 20%-66% (w/v) sucrose gradient for 5 min. **(D,E)** XPS spectra of f10 and f30 GO fractions from density gradient ultracentrifugal rate separation in 20%-66% (w/v) sucrose gradient for 5 min. **(F)** Fluorescence (λex = 400 nm) spectra of GO in different fractions from density gradient ultracentrifugal rate separation in 20%-66% (w/v) sucrose gradient for 5 min. Reproduced with permission from Sun, X., Luo, D., Liu, J. & Evans, D. G. Monodisperse chemically modified graphene obtained by density gradient ultracentrifugal rate separation. ACS Nano (2010) doi: 10.1021/nn1000386. Copyright (2010) American Chemical Society.

Centrifugation is one of the most widely used techniques for separating mixtures by size and density. This technique relies on the different sedimentation rate of a material as a function of its size, shape, and buoyant density, but also of the density and viscosity of the centrifugal medium and the rotor speed. The rotor spin results in a centrifugal force that pushes the sample particles downwards. The sedimentation rate of each particle in the sample is dependent on the physical properties of the particles, the viscosity of the medium, and the centrifugal force applied. For equal centrifugal force and viscosity of the medium, the sedimentation rate of a particle is related to its size (molecular weight) and the difference between the density of the particles and the density of the solution. Therefore, particles of different size, or density, sediment at different speeds and separate. Recently, because of its high performance, scalable production capacity, and lack of nanoparticle aggregation, direct dimensional separation in the liquid phase by centrifugation has proven to be an effective process for the separation of gold particles, carbon nanotubes, and graphene materials (Green and Hersam, 2009; Hároz et al., 2010; Qiu and Mao, 2011). There are two basic types of centrifugal separation, differential pelleting and density gradient. In the differential centrifugation method, the materials to be separated are divided into several fractions according to their size by varying the centrifugal forces applied. The centrifuge tube is loaded with a uniform mixture of the sample solution and centrifuged at low speed. At the end of the cycle, two fractions are obtained: a pellet containing the largest sedimented material and a supernatant solution of non-sedimented material. The two fractions are extracted by decanting the supernatant solution from the pellet. The smaller material in the supernatant is further differentiated according to its size by gradually increasing the speed of each cycle. Alternatively, the starting solution can be centrifuged at high speed by separating the small particles in the supernatant from the large particles in the sediment, and then the precipitate can be dimensionally differentiated by reducing the centrifugal forces applied. This separation method is applied to GO flakes prepared with the modified Hummers method (Chen et al., 2013b). The aqueous suspension of GO (0.1 mg mL^–1^) obtained by ultrasonication and centrifugation from Hummers methods was processed by successive steps of centrifugation at decreasing speed. In each centrifuge cycle, the average size of the GO nanosheets in the supernatant decreases because the larger flakes sediment at a higher speed as opposed to the applied acceleration. AFM images of the three aqueous suspensions separated by this method, reveal flakes with a quite uniform lateral size distribution, and different average lateral size from 260 nm, for the supernatant obtained at high centrifugation rates, to 977 nm, for the one obtained at lower rates. When considering the dimensional separation of GO by centrifugation, it must always be considered that the theory of sedimentation, based on the assumption of spherical and homogeneous sediment particles (Stokes law) may have limitations when applied to an inhomogeneous and two-dimensional material. Huang and Yuan (2016) carefully characterized aqueous suspensions of GO separated by differential gradient centrifugation, demonstrating that the sedimentation process of GO in aqueous solution does not depend only on the lateral dimensions, but especially on the degree of oxidation and the interlayer distance between the graphene layers in GO. In particular, a high degree of oxidation and a larger *d-*spacing increase the hydration level of the sheets and they sediment more slowly due to the higher viscous resistance (Huang and Yuan, 2016). Another separation method is density gradient centrifugation in which the components of a sample are placed on the surface of a vertical column of liquid with increasing density from top to bottom and then centrifuged. The two main types of density gradient centrifugation are rate-zonal separation and isopycnic separation. In the isopycnic technique, the sample has a density between the lowest and the highest density of the column gradient. The particles migrate through the solvent gradient, during centrifugation, until they reach the point where their buoyancy density is equal to that of the gradient. This is known as the isopycnic point or isodense position. This separation method based on balancing the density of the colloidal material with that of a support medium was applied to separate carbon nanotubes by diameter/chirality/wall thickness (Arnold et al., 2005; Arnold et al., 2006; Wei et al., 2008; Green et al., 2009) and to separate GO sheets by the number of layers using a density gradient formed by aqueous solutions of non-ionic gradient medium, iodixanol. GO-iodixanol complexes have buoyancy densities that vary with the thickness of the encapsulated GO sheets and can be separated regardless of their lateral size (Li et al., 2013). In rate zonal centrifugation, instead, the gradient has a lower density throughout the tube to ensure that the distance a particle travels through the gradient depends exclusively on the diameter of the particle. Larger particles will be able to travel to the lower layer because they are more massive. The higher mass allows particles to travel through layers with a higher viscosity, while the smaller particles will stay at the top because they do not have the mass to travel through the more viscous layers. In this way, the particles that sediment faster are not contaminated by the slower particles as in differential centrifugation. The first-rate zonal separation of GO nano-sheets was reported in Sun et al. (2008). In this paper, the authors explore cellular imaging and drug delivery properties of nGO sheets prepared by the modified Hummer method from expanded graphite and functionalized with PEG to enhance solubility and stability in salts and cell solutions. The mixture containing functionalized GO sheets is then subjected to 2.5 h of centrifugation at ∼ 30000 *g* in an iodixanol gradient obtaining multiple layers of different size PEG- GO sheets along with the centrifuge tube. AFM images of the fractions collected layer by layer show that the flake size increases monotonically along the centrifuge tube and in each fraction, most of the flakes have a uniform lateral size. This method was subsequently optimized and applied to pure GO (Bidram et al., 2016). Using a simple sucrose gradient, Sun et al. (2010) demonstrated that by adjusting the separation parameters, including centrifugation time and density gradient profile it is possible to obtain GO sheets with targeted size and degree of oxidation. In their method, a freshly prepared GO suspension was layered on top of the density gradient (20-66% gradient) and centrifuged at 50K rpm for 15 min. After centrifugation, GO sheets were separated into different zones along the centrifuge tube, AFM characterization indicated that sampling along the centrifuge tube yielded GO sheets of increasing size from 40 (upper fractions) to 450 nm (down fractions) while TEM characterization showed a single layer structure for GO sheets found at the top of centrifuge tube and a multilayer structure for those at the bottom of tube. These results are coherent with the sedimentation behavior of GO sheets, the thick and heavy multilayered structures sediment faster than single layered ones, allowing single layered fractions to separate according to size. Reducing the centrifugation time from 15 to 5 min results in fractions separated in terms of degree of oxidation. This result was confirmed by using various characterization techniques (Figure 10). Calling f5, 10, 15, 20, 30 the fractions collected from top to bottom in the centrifuge tube, Figure 10A shows absorption measurements in the UV-vis range after curves normalization to the absorbance peak relative to a π-electron plasmon excitation of graphitic carbon at 230 nm. GO sheets in upper fractions had much lower absorbance in the visible range, 400-800 nm, while the visible absorbance of lower fractions increased significantly. “Pristine” graphene made by intercalation and exfoliation without oxidation showed a ratio of absorbance intensity at 400 nm to that at 800 nm (Abs400 nm/Abs800 nm) near to 1.5:1 (Li et al., 2008), while fully oxidized GO showed a ratio near to 4.5:1. Similar results are obtained in the separated fractions with the density gradient ultracentrifugal rate separation method (Figure 10B), indicating that GO sheets were separated in terms of degree of oxidation, with less functionalized graphene sheets being distributed in higher fraction numbers. This was further confirmed by the red shift of the UV absorption peak from ca. 230 nm for f5 to 260 nm for f30 (Figure 10C) such a red shift has previously been reported when the degree of reduction of GO is increased (Wang et al., 2008). The C-O peak (at ca. 286.5 eV) in XPS spectrum of f30 fraction (Figure 10D) is much weaker than that in the f10 fraction (Figure 10E) indicating a lower number of oxygenated functional groups present in the lower fractions and therefore a low degree of oxidation. The high fluorescence intensity of the f5 fraction (Figure 10F) is very interesting for drug delivery and cellular imaging applications. Fluorescence is consistent with the presence of GO sheets with extremely small size and a high degree of oxidation.

#### Flocculation by Solvent

The colloidal stability of GO sheets in aqueous solution resulted from the competition between the dispersive electrostatic repulsive interactions between the sheets, arising from the ionized carboxyl groups on the edges, and the attractive Van der Waals face-to-face interactions that promote aggregation. The increased colloidal stability of GO in aqueous solution is due to a combination of increased electrostatic repulsion, decreased overlap areas, and decreased probability of overlapping. Therefore, in an aqueous medium with the same pH value, GO sheets with smaller size could have a higher solubility than larger sheets because they exhibit a high edge to area ratio and a higher density of ionized carboxylic groups. In addition, the solubility of GO sheets in water is a function of the pH of the dispersion, as the pH of the medium decreases, the carboxyl groups on the edges become protonated, the repulsion forces decrease, the Van der Waals attraction forces prevail and the colloids precipitate. Wang X. et al. (2011) used this pH dependence to selectively precipitate large quantities of GO sheets mostly larger than 40 μm^2^ and narrow size distribution raising the pH of the aqueous dispersion to 4 with HCl 1M. Instead, Zhang et al. (2015) use the protonation in organic solvents (ethanol, tetrahydrofuran) of carboxyl groups on the edges of GO sheets to selectively precipitate large GO sheets according to their size. Starting from the consideration that GO sheets with smaller lateral dimensions have greater colloidal stability, Hu et al. (2015) reconsidered the general work-up procedure of classical GO synthesis. Usually, after quenching with distilled water containing a small amount of H_2_O_2_, the reaction product is filtered and the precipitate further purified. The authors have proven instead that the strongly acidic filtrate (pH ∼ 1) recovered from filtration contains unprecipitated nGO. The nGO present in the filtrate undergoes fast aggregation and forms stable floccules by gradual addition at room temperature of a 1M KOH or NaOH solution until the pH value increases to ∼1.7 (Hu et al., 2015). The sediment, isolated and characterized, contained nGO flakes with a multi-layer structure [thickness of about 3.5 nm (4-5 layers)] and narrow size distribution with an average size of less than 90 nm.

## Nano Graphene Oxide in Cancer Therapy

The intrinsic properties of nGO make this material very interesting in the field of biomedicine. The oxygen atoms chemically bonded to the carbon lattice by treatment with strong oxidizing agents in an acidic environment are defects introduced in the ideal graphite plane, but they provide the GO with unique properties such as hydrophilicity, i.e., the ability to dissolve and to form stable colloidal solutions in water and some low molecular weight alcohols. The oxygenated groups present on the surface of the GO and the nanometric dimensions are not enough to balance the screening of the electrostatic charges when the nGO is in biological media, consequently, the nGO tends to aggregate in physiological solutions with salts and proteins (Muñoz et al., 2019). The surface functionalization of GO increases solubility under physiological conditions and opens the scenario to a wide range of biomedical applications. In general, the chemical functionalization of the GO may be of covalent or non-covalent type. Covalent functionalization involves the conversion of the functional groups present on the GO and/or the chemical bond between the functional groups and external species such as small molecules, polymers, and inorganic particles. Non-covalent functionalization, on the other hand, exploits the large, atomically flat surface of GO as an anchor for other chemical species through secondary interactions such as Van der Waals forces and π-π interactions (Lonkar et al., 2015).

### Drug Delivery Application in Cancer Therapy

**FIGURE 11:**
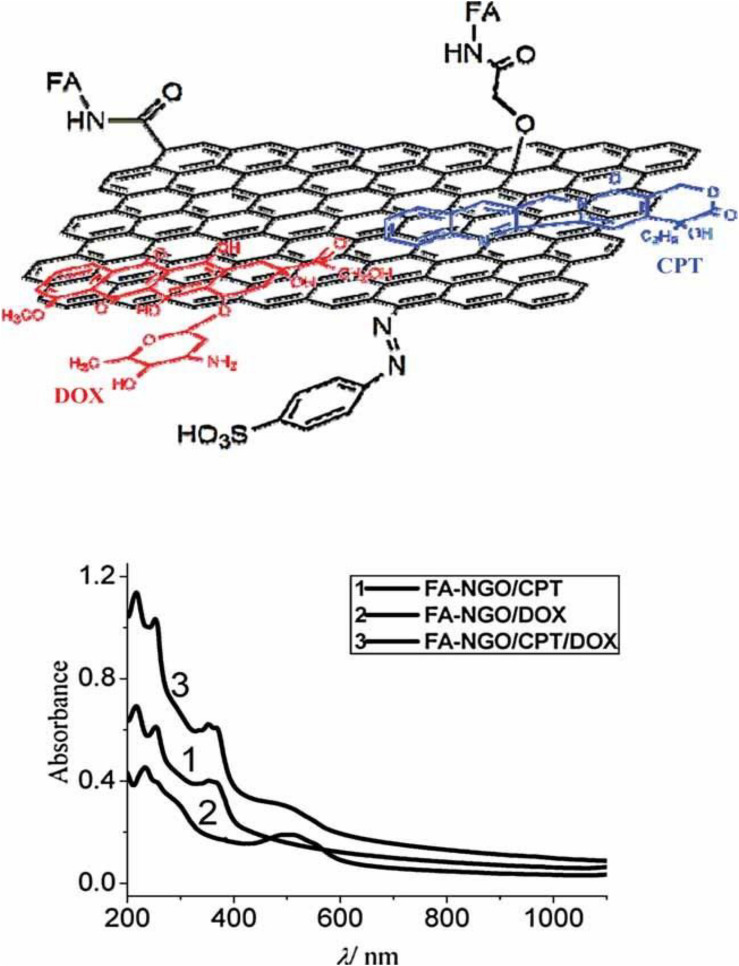
**(A)** Schematic representation of the simultaneous loading of doxorubicin and campotecin on both sides of the GO sheets. **(B)** UV/VIS spectra of DOX, CPT, and a mixture of DOX and CPT each loaded onto FA–NGO. Reproduced from Zhang, L., Xia, J., Zhao, Q., Liu, L. & Zhang, Z. Functional graphene oxide as a nanocarrier for controlled loading and targeted delivery of mixed anticancer drugs. Small (2010) doi: 10.1002/smll.200901680.

Graphene oxide can be used as an efficient nanocarrier for the loading and transport of water-insoluble aromatic molecules using non-covalent interactions. Approximately 50% of GO carbon atoms are sp^2^ carbons and therefore can interact with conjugated π molecules. After the pioneering study by Dai and collaborators Liu et al. (2008), many other research groups have confirmed the excellent drug loading ability of the nGO. In this context Zhang et al. (2010) first demonstrated the ability of nGO to load simultaneously two drugs [doxorubicin (DOX) and campotecin (CPT)] with synergistic action and a loading efficacy comparable to the loading of a single drug. This approach is very advantageous because it allows to the reduction of multi-drug resistance to anticancer drugs, the phenomenon that occurs when cancer cells develop resistance to anticancer drugs with different structures and mechanisms of action (Szakács et al., 2006). In this work, the nGO is covalently modified with folic acid molecules for selective targeting of folate receptors overexpressed by cancer cells, and two aromatic anticancer drugs were loaded on both sides of the graphene sheets through π–π stacking and hydrophobic non-covalent interactions (Figure 11A). The amount of drug-loaded was estimated by UV/Vis spectroscopy. The simultaneous presence of absorption peaks at ∼490 nm (characteristic of DOX) and ∼365 nm (characteristic of CPT) suggested that both DOX and CPT were loaded onto the nGO -folic acid platform (Figure 11B). This system displayed therapeutic efficacy in killing cells from the human breast cancer cell line (MCF-7).

**FIGURE 12:**
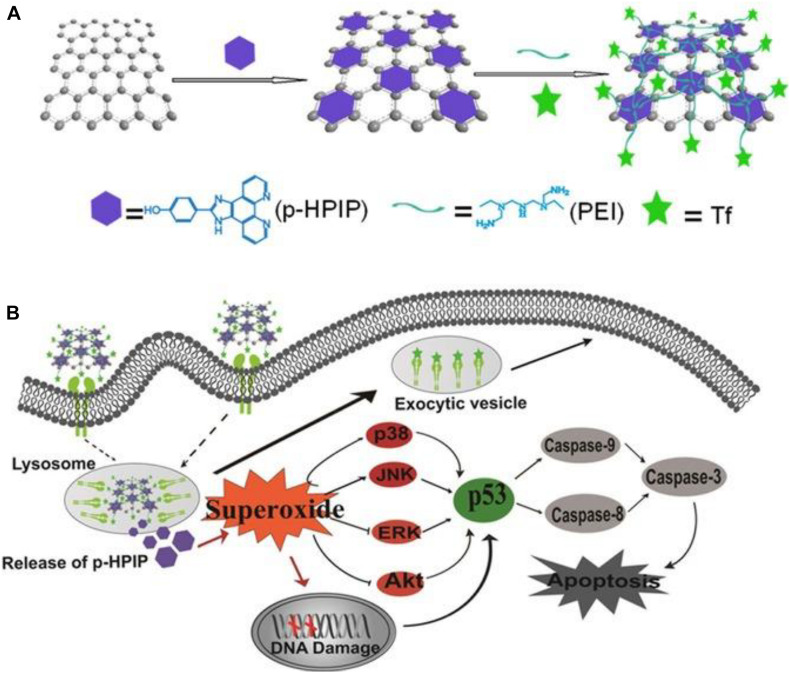
**(A)** Synthetic scheme for the double functionalization of the nGO. **(B)** Schematic illustration of the activation intracellular apoptotic signaling pathways by Tf-NGO@HPIP. Reproduced from Zhou, B., Huang, Y., Yang, F., Zheng, W. & Chen, T. Dual-Functional Nanographene Oxide as Cancer-Targeted Drug-Delivery System to Selectively Induce Cancer-Cell Apoptosis. Chem. - An Asian J. (2016) doi: 10.1002/asia.201501277.

The strategy of overcoming MDR by loading multiple drugs with different actions on the same nanocarrier is frequently used and the nGO is one of the most flexible systems for this purpose. The planar carbon atoms structure allows combining, through non-covalent interactions, multiple hydrophobic drugs with high loading efficiency. **Deb et al. (2018)** studied the synergistic effect against breast cancer of two anti-cancer drugs, camptothecin (CPT) and 3,3’ diindolylmethane (DIM), co-loaded on a nano biocomposite (GO-CS-FA-CPT-DIM) of GO functionalized with chitosan (CT) and decorated with folic acid (FA), as a targeting moiety (Deb et al., 2018). The *in vitro* tests showed that the anticancer activity of GO-CS-FA-CPT-DIM was higher in comparison to the free CPT probably because of the different mechanisms of action employed by CPT and DIM. In addition, *in vivo* tests revealed a reduction in common side effects of CPT when administered free, such as gastrointestinal diseases, disruption of renal function, cumulative hematological toxicity, and liver inflammations. Another combination of chemotherapy drugs with synergistic effect loaded on GO was contemporaneously tested by Tiwari et al. (2019). They loaded two widely used anticancer chemotherapy drugs, quercetin, and gefitinib on a GO larger than 100 nm grafted with polyvinylpyrrolidone (PVD), a highly hydrophilic and biologically compatible polymer, and investigated the loading and cancer cells cytotoxicity of two individual drug systems and combined system in ovarian cancer cells PA-1 compared to ovarian epithelial cells IOSE-364. They founded that the release rate of the two drugs combined on the GO nanocarrier coated with PVP in phosphate-buffered saline (PBS) solution was higher than that of individual drugs on the same system. They attributed the better release rate to intermolecular interactions, such as π-π stacking, and to the strong inter- and intra-molecular hydrogen bonds between the electronegative atoms present in the structure of the drug molecules and the carrier. In addition, their results showed that both the systems with single drugs and the system with combined drugs had no significant cytotoxic effects on the IOSE cells, while high toxicity was detected on ovarian cancer cells PA-1, and the combined system showed the highest toxicity at the same concentration. **Bullo et al. (2019)** designed an anticancer nanocomposite of nGO coated with polyethylene glycol and loaded with two anticancer drugs; protocatechuic acid (PCA) and chlorogenic acid (CA). The DLS measurements showed that the final nanocomposite had a narrow distribution, in the range of 10-40 nm with a median value of 8 nm. This nano-formulation showed strong anticancer activity against liver cancer, HepG2, and colon cancer, HT29 cells compared to free drugs. Covalent functionalization includes a variety of reactions as esterification, amidation, click chemistry, nitrene chemistry, and radical addition (Layek and Nandi, 2013) and is widely used for drug loading, coating the nGO with biocompatible polymers, or bonding targeting motives. According to widely agreed Lerf and Klinowski’s model, GO sheets have chemically reactive oxygenated functions, such as carboxylic acid groups at the edges and epoxy and hydroxylic groups on the basal planes (He et al., 1998). Although there are examples of functionalization of the hydroxyl and epoxy groups located in the basal plane of the GO (Layek et al., 2010**;** Sydlik and Swager, 2013), the chemistry of GO is dominated by the chemistry of the carboxylic and carbonylic functional groups located at the edges. Most approaches exploit the protocols of carboxylic acid conversion reactions into esters or amides (Zhang X. et al., 2009; Yu et al., 2010). The introduction of substituted amines is one of the most common covalent functionalization methods and the final products are designed for various applications. An example of the use of GO functionalized amine (GO-NH_2_) for biological applications is provided by the work of Singh et al. (2012). They synthesized GO-NH_2_ by activation of GO carboxylic acid groups with thionyl chloride in dimethylformamide and subsequent reaction with sodium azide and demonstrated that it was a safer alternative for biomedical applications, compared to oxygenated derivatives. It is known that GO, when administered intravenously in mice, has the potential to cause a strong aggregation response in platelets on a scale comparable to that caused by thrombin, one of the most powerful physiological platelet agonists, and triggers extensive pulmonary thromboembolism, consistent with the prothrombotic nature of this material (Singh et al., 2011). *In vivo* and *in vitro* tests showed that modified amine GO, being positively charged, was more biocompatible than GO, had no stimulating action toward platelets and does not induce pulmonary thromboembolism in mice. Wojtoniszak et al. (2013) covalently bonded GO with methotrexate (MTX), a folic acid analog used as a chemotherapeutic agent via amide binding. Wu et al. (2014) used the same chemical route to functionalize a nano-size GO with hyaluronic acid (HA), a naturally occurring polysaccharide with excellent physicochemical properties, such as biodegradability, biocompatibility, and non-immunogenicity. The obtained nGO-HA system showed a high loading capacity of the anti-tumor drug DOX and high cytotoxicity to cervical cancer cells. The *in vivo* toxicity studies showed very low cytotoxicity, good blood compatibility, and no evident toxic effects in mice. GO–HA/DOX could selectively accumulate in the malignant tumor issues by receptor-mediated endocytosis and inhibit tumor growth. As discussed extensively earlier, one way to increase nanocarrier accumulation at the tumor site is to bond ligand at the nanocarrier surface to selectively recognize specific molecules that are over-expressed by tumor cells and tissues, but are not present in healthy cells and tissues. Li J. et al. (2018) presented a dual-targeting platform of GO with high stability and drug loading capacity for the inhibition of pulmonary metastasis of breast cancer. This platform was based on a GO modified with folic acid and heparin via a polyethyleneimine linker. Heparin with a quantity of hydrophilic carboxylic and sulphonated groups maintained the GO surface negatively charged, thus avoiding rapid elimination from circulation and acting as a potential targeting material because it could bind competitively to the receptor for advanced glycation end products-mediated expression of malignant cells. The *in vivo* assays indicated that this dual targeting system could not only inhibit the *in situ* tissue growth, but also suppress the pulmonary metastasis. Pham et al. obtained a GO with lateral size less than 100 nm by ultrasonication of the graphite oxide flakes obtained by the Hummer’s method and chemically functionalized it with alendronate (AL), a second-generation bisphosphonate approved by FDA to treat tumor-associated hypercalcemia and several bone-related diseases for the treatment of bone metastasis in advanced breast cancer (Pham et al., 2019). The *in vivo* tests showed that the alendronate functionalization was able to increase NGO accumulation in the skeletal system and enhanced *in vivo* retention times after intravenous administration. In addition, the nanosheets preferentially accumulated in bone bearing tumors and not in healthy bones due to the tumor microenvironment. Bone surfaces are normally covered by cells and organic matrix, which might reduce AL binding, whereas bone remodeling during osteolytic lesion development leads to the digestion of bone organic matrix and cell detachment. GO has also been explored to implement drug delivery systems able to respond intelligently to the tumor microenvironment. As is well known, the extracellular pH of tumor tissue is often acidic due to acid metabolites caused by anaerobic glycolysis in hypoxia. This pH difference can be utilized to achieve a targeted release of the drug. For this purpose, Depan et al. (2011) synthesized a novel drug carrier based on GO by attaching DOX to GO via strong π–π stacking interaction, followed by encapsulation of GO with folic acid conjugated chitosan. The hydrophilicity and cationic nature of chitosan enhanced the stability of the nanocarrier system in the aqueous medium. The loading and release of DOX are strongly pH-dependent and compared to pH 7.4, the nano-hybrid system exhibited higher drug release at pH 5.3, which is ascribed due to the reduced interaction between DOX and drug carrier. Zhou T. et al. (2014) exploited citraconic anhydride-functionalized poly(allylamine) (PAH-Cit), a common charge-reversal polyelectrolyte, which can be readily converted back to cationic poly(allylamine) by amide hydrolysis upon exposure to mild acidic environments, such as those found within late endosomes and lysosomes. They synthesized the charge-reversal polyelectrolyte and loaded it onto GO sheets by electrostatic interaction, then bonded the DOX to anionic PAH-Cit by covalent linkage of carboxyl group with amino group. The release mechanism of the drug involveed the conversion of PAH-Cit into cationic poly(allylamine) as a result of endocytosis and exposure to acidic endosome or lysosome environments. Lv et al. (2016) developed a multifunctional GO as a drug carrier targeting to hepatocarcinoma cells. They first modified the GO surface with polyethyleneimine (PEI), then they derivatized the PEI-NH_2_ groups with fluorescein isothiocyanate (FI), as imaging dye, and polyethylene glycol (PEG)-linked lactobionic acid (LA), as targeting ligand, and acetylated the remaining terminal amines of the PEI. The formed carrier, loaded with DOX through π–π stacking interactions was water soluble and displayed a pH-responsive DOX release behavior with a faster DOX release rate at pH 5.8 than that of the physiological pH. Another important feature of this carrier was its good cell viability and the ability to specifically target hepatocarcinoma cells overexpressing asialoglycoprotein receptors. An analogous pH responsive drug delivery system based on GO functionalized with carboxymethyl chitosan (CMC), fluorescein isothiocyanate and lactobionic acid (LA) was developed by Pan et al. (2016). A nGO with lateral size of 40 nm was double-functionalized with 2-(4-hydroxyphenyl)imidazo[4,5-f] [1,10]phenanthroline (p-HPIP), an excellent intercalating ligand in DNA-binding, polyethylenimine (PEI), to improve the stability of NGO-based nanosystem under water/physiological conditions and transferrin (Tf) as a surface decorator by Zhou et al. (2016)**;** Figure 12A. This nano-system (Tf-NGO@HPIP) was internalized through receptor-mediated endocytosis and triggered pH-dependent drug release in acidic environments and in presence of cellular enzymes. Moreover, it showed enhanced cytotoxicity toward cancer cells by triggering cell apoptosis through the overproduction of intracellular superoxides (Figure 12B).

**FIGURE 13:**
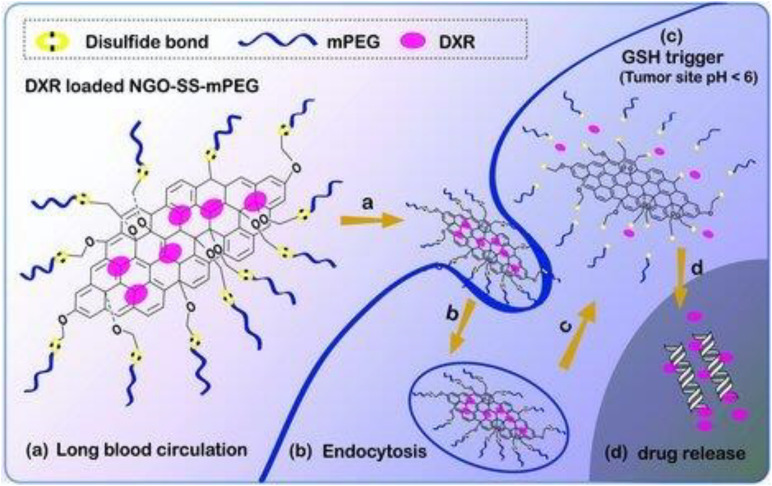
Schematic diagram illustrating the fate of redox-sensitive GO-based nanocarrier. When PEG-coated nGO **(a)** with disulfide bridges is endocytosed in tumor cells by EPR effect **(b)**, the high concentration of GSH at the tumor site triggers PEG detachment **(c)** and the rapid release of the loaded drug **(d)**. Reproduced from Wen, H. et al. Engineered redox-responsive PEG detachment mechanism in PEGylated nano-graphene oxide for intracellular drug delivery. Small (2012) doi: 10.1002/smll.201101613.

Another research group, instead, exploited the high concentration of glutathione (GSH) present in the cytosol of tumor cells to trigger the release of the drug from a nanocarrier consisting of GO coated with a newly synthesized PEG cross-linked via disulfide bonds and loaded with DOX hydrochloride (Wen et al., 2012). The PEG coating improved the stability and circulation time of the nanocarrier, while the presence of disulfide bonds allowed the rapid release of the drug through the exchange reaction of thiol ligands by cellular GSH. In the presence of high GSH concentration, PEG-coated NGO was internalized via endocytosis thereby initiating rapid disulfide cleavage of a stabilizing PEG shell, which initiates the rapid release of encapsulated payload (Figure 13). The confocal laser scanning microscopy and flow cytometric analyses demonstrated the pharmacological efficacy of the intracellular release of doxorubicin hydrochloride from functionalized nanocarrier in the presence of elevated GSH concentrations.

**FIGURE 14:**
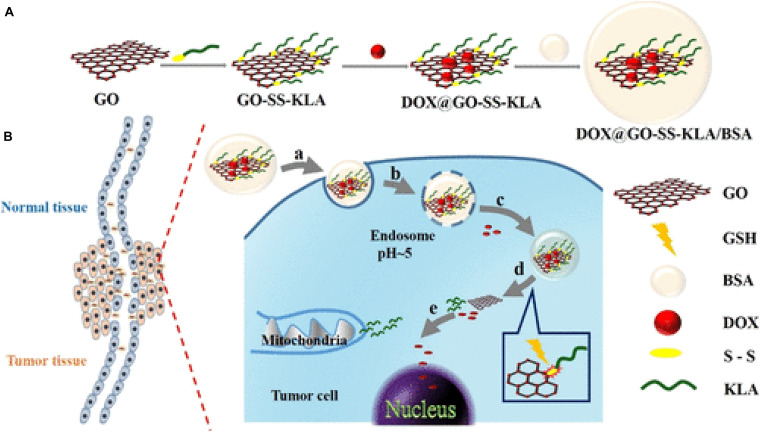
**(A)** Synthetic route for GO - proapoptotic peptide- DOX double sensitive nanocarrier. **(B)** action mechanism after nanocarrier accumulation in tumor tissue by the EPR effect. Reproduced with permission from Zhang, J. et al. Dual-Sensitive Graphene Oxide Loaded with Proapoptotic Peptides and Anticancer Drugs for Cancer Synergetic Therapy. Langmuir (2019) doi: 10.1021/acs.langmuir.9b00611. Copyright (2019) American Chemical Society.

Zhao et al. (2014) oxidized for 36 h a graphite pre-treated with P_2_O_5_ and K_2_S_2_O_8_ to obtain nGO with nearly spherical shaped and monodisperse size of 43.36 ± 8.42 nm. To increase the half-life and bypass recognition and clearance by the reticuloendothelial system (RES), the GO nano-spheres were coated with a PEG synthesized to be responsive to the tumor reducing environment. This strategy involved the PEGylation of alginate (ALG), to obtain ALG-PEG, and then a conjugation of cytamine (Cy) to ALG-PEG by the amidation between the -NH_2_ group of Cy and the -COOH groups of ALG. The Cy-ALG-PEG polymer was grafted onto the surface of the nGO via a reduction-sensitive disulfide bond, so the nanocarrier was responsive to cleave the disulfide bond to detach the Cy-ALG-PEG polymer moieties in reducing condition. The near-spherical shape and nanometric dimensions of the carrier also allow for high DOX loading capacity and excellent encapsulation efficiency. The same research group also investigated the functionalization of GO to obtain systems responsive to more than one stimulus (Zhao et al., 2015) through a PEGylate GO functionalized with cytamine. The authors studied the release of DOX *in vitro* simulating pH and glutathione concentration levels under physiological conditions (pH 7.4 and [GSH] 10 μM) and in the tumor microenvironment (pH 5.0-5.5 and [GSH] up to 10 mM) and founded that the initial DOX release was 6-fold faster at pH 5.0 in the presence of 10 mM than at pH 7.4 in the presence of 10 μM GSH. As the simultaneous loading of several drugs with different mechanisms of action on the same nanocarrier, obtaining a nanocarrier sensitive to double/multiple endogenous stimuli is also a way to increase therapeutic efficacy. Recently, Zhang J. et al. (2019) designed and constructed a dual-sensitive cancer combination treatment system utilizing GO to load proapoptotic peptide (KLA) and anticancer drugs. The proapoptotic peptide is an amino acid sequence of lysine (K), leucine (L), and alanine (A), (KLAKLAK)_2_, that can induce mitochondrial-dependent apoptosis while remaining relatively non-toxic extracellularly. The GO was firstly modified with (3-mercaptopropyl)-trimethoxysilane to obtain GO-SH, then converted in GO-SSNH_2_ by reaction with S-(2-aminoethylthio)-2-thiopyridine hydrochloride and finally, alkyne-modified disulfide-functionalized GO (GO-SS-alkyne) was obtained by reaction with propargyl bromide. The proapoptotic peptide was synthesized by employing a standard Fmoc chemistry through the solid-phase peptide synthesis and terminated with azide group (-N_3_) and connect onto GO-SS-alkyne by azide-alkyne click chemistry reaction. Then, the aromatic anticancer drug DOX was loaded on GO by π-π conjugation and hydrogen bonding interactions and bovine serum albumin (BSA) was used to coat the GO carrier to enhance the stability of the system and the hydrophilicity of the GO carrier after drug loading (Figure 14A). The action mechanism of the double sensitive nanocarrier is schematically illustrated in Figure 14B. After accumulation in the tumor tissues by the EPR effect, the nanocomposite penetrated the cell membrane and was uptaken by the tumor cells (a), the low pH triggered the release of DOX in the endosomes (b,c), while the high concentration of GSH induced the cleavage of the KLA from the surface of the GO by the scission of the disulphide bonds (d). The free peptide and the Dox showed a synergistic therapeutic effect (e).

### Theranostic Application in Cancer Therapy

**FIGURE 15:**
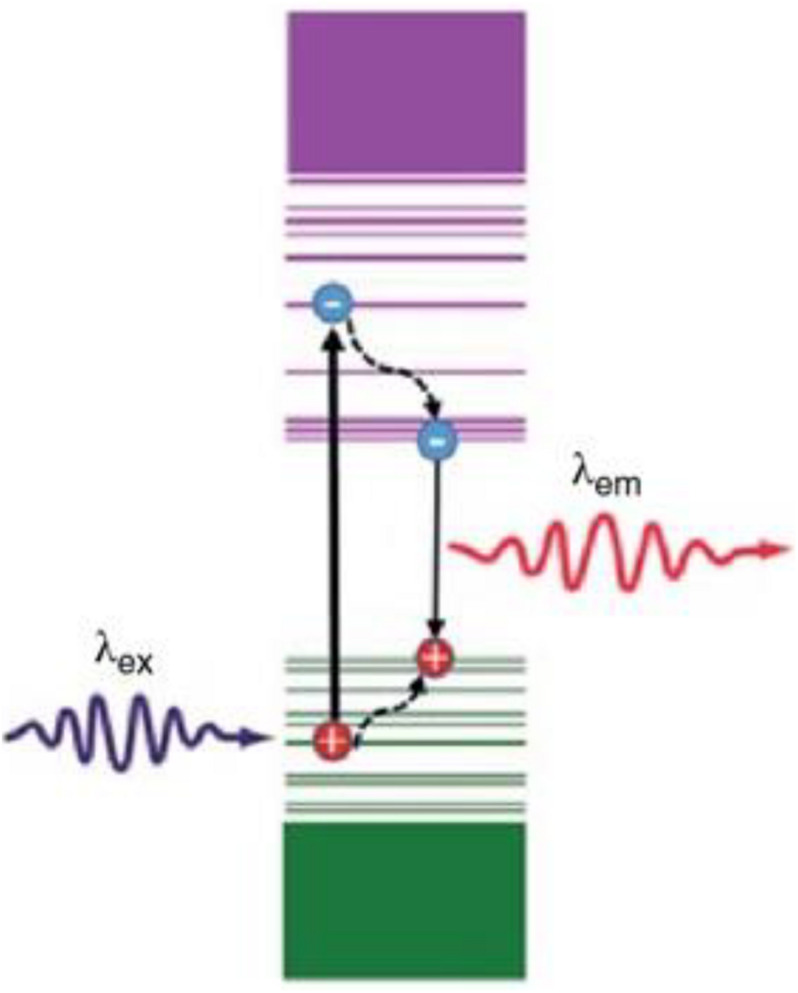
Illustration of photoluminescence mechanism of GO.

As described above, GO exhibits size-dependent photoluminescence. The photoluminescence of GO is due to the functionalization of the carbon lattice, which induces the opening of the energy gap. When the absorption of an incident photon occurs, an electron is promoted to a higher energy level orbital, leaving a positively charged hole below (Figure 15). This electron-hole pair, created for the absorption of a photon is called an exciton.

**FIGURE 16:**
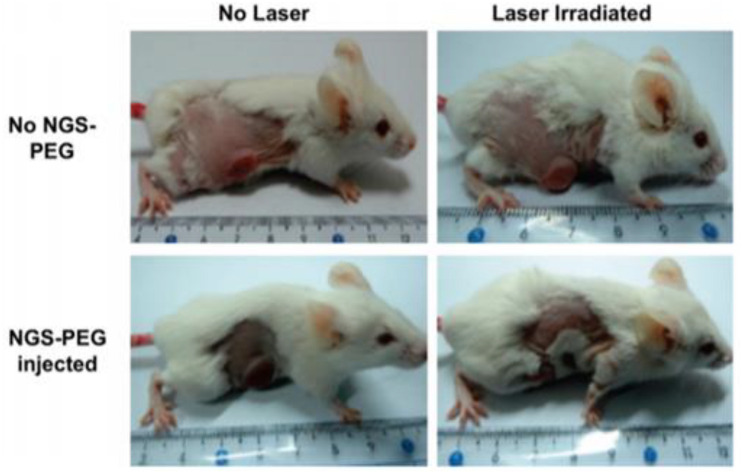
representative photos of tumor ablation in mice after photothermal treatment. Reproduced with permission from Yang, K. et al. Graphene in mice: Ultrahigh *in vivo* tumor uptake and efficient photothermal therapy. Nano Lett. (2010) doi: 10.1021/nl100996u. Copyright (2010) American Chemical Society.

The creation of an exciton is followed by the non-radiative decay of the electron at the lower energy empty molecular orbital (LUMO) and the hole at the higher energy occupied orbital (HOMO). This process is then followed by radiative recombination of the exciton that generates the emission of a photon with lower energy than the incident photon. The efficiency of this process is described by the quantum fluorescence yield, i.e., the ratio between absorbed and emitted photons, which, for the GO, is reported to be 0.02-0.5% due to the electron-hole recombination through non-radiative processes. Among the non-radiative processes involved, conversion to thermal energy is used for photothermal therapy of GO. Photothermal therapy uses an optical absorbing agent to generate heat as a result of light radiation, producing a temperature increase that kills the cancer cells (Yang et al., 2013). Electromagnetic radiation with a wavelength between 650 and 900 nm (NIR) is very interesting for medical applications because in this window absorption by the skin and tissues is minimal and penetration is intense (Weissleder, 2001). The results of GO as a photothermal agent for tumor ablation are presented for the first time in Yang et al. (2010). In this study, single or double-layer GO nano-sheets with lateral dimensions between 10 and 50 nm coated with amine-terminated six-arm branched PEG (NGS-PEG) were used. To verify the potential in photothermal therapy, an aqueous dispersion of NGS-PEG at a concentration of 0.5 mg/mL was irradiated with a NIR laser at 808 nm with a power density of 2W cm^–2^, using water as a control. In contrast to the water sample, the NGS-PEG solution showed a rapid increase in T. The *in vivo* behavior of the PEGylated nGO sheet labeled with a fluorescent dye was studied in tumor-bearing mice using *in vivo* fluorescence imaging. Polyethylene glycol-conjugated nanofold showed high passive tumor accumulation due to the EPR effect in several different tumor models and low retention in the reticuloendothelial systems. Fluorescence measures showed that the system was widely distributed throughout the body 30 min after injection, but tended to accumulate in the tumor over time, particularly 24h after injection there was an accumulation in the tumor with relatively low signals in other parts of the body. The dye or PEGylated dye at the same concentration, used as a control, was eliminated after a few hours (renal clearance). In the same study, to explore the effects of photothermal therapy *in vivo* a NIR (808 nm) laser at a power density of 2 W cm^–2^ was used to ablate tumors bearing the functional nanosheets. The superficial temperature of tumors in mice injected with NGS reaches about 50°C after laser irradiation, while the T of the control increases by about 2°C. All tumors on mice treated with NGS disappeared one day after treatment, leaving black scar tissue that went away one week after treatment (Figure 16), no reappearance of the tumor was observed in the 40 days following treatment. In contrast, the three control groups all exhibited rapid tumor growth, indicating that NIR radiation alone did not affect tumor growth. Moreover, mice in the control groups showed an average life span of about 16 days, while mice injected with NGS survived over 40 days.

**FIGURE 17:**
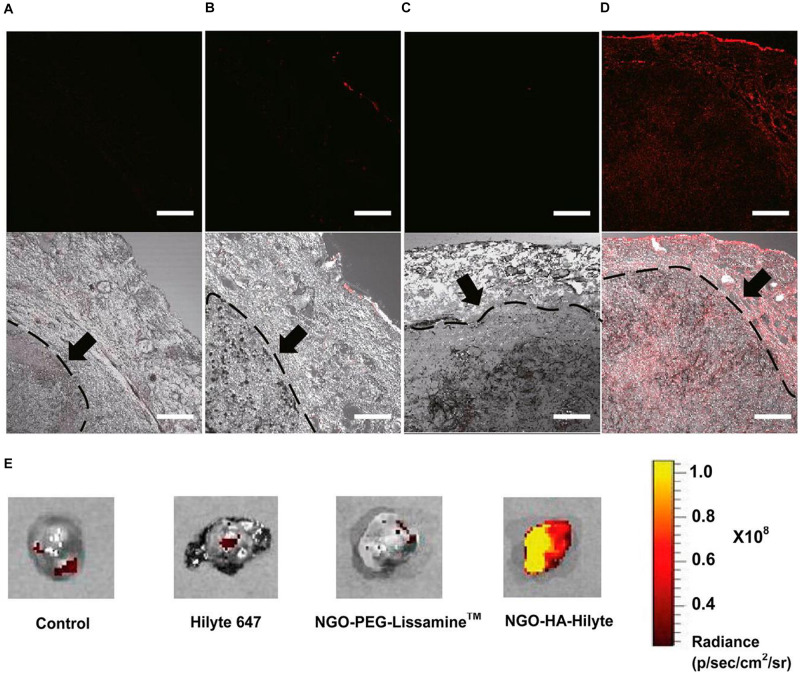
Confocal microscopic images for the *in vitro* transdermal delivery of panels **(A)** PBS, **(B)** Hilyte647 dye only, **(C)** nGO -poly(ethylene glycol)-Lissamine (fluorescent dye) and **(D)** NGO-hyaluronic acid-Hilyte647 (fluorescent dye) (scale bar = 200 μ m). Arrows indicate the tumor regions. **(E)** *Ex vivo* bioimaging of dissected tumor tissues. Reproduced with the permission from Jung, H. S. et al. Nanographene oxide-hyaluronic acid conjugate for photothermal ablation therapy of skin cancer. ACS Nano (2014) doi: 10.1021/nn405383a. Copyright (2014) American Chemical Society.

Kurapati and Raichur (2013) synthesized GO–poly(allylamine hydrochloride) (PAH) composite capsules using dextran sulfate (DS) doped calcium carbonate [CaCO_3_ (DS)] as a sacrificial template and studied the capsule breaking mechanism and the releasing of encapsulated anticancer drug DOX upon NIR-laser (1064 nm) irradiation for different periods and power of the laser. The optical microscopy and low and high magnification transmission electron microscopy images showed that the breakage of the capsules by irradiation with NIR light began with a small hole and then extended as exposure time and laser power increased. This kind of breaking is known in the literature as a “point-wise opening” and has been reported for gold nanoparticles (Bédard et al., 2008). As reported earlier, NIR-laser irradiation of GO resulted in the generation of excitons, which decay into heat and produced strong heating of the surrounding environment. GO has superior thermal conductivity, specific heat capacity, and NIR-absorption compared to gold nanoparticles, and upon irradiation of the NIR-laser (30 mW) for 45 s, the capsule suspension temperature was increased from 25°C to 40°C due to local heating. Similarly, the amount of DOX released increased, in comparison to the control, as the breakage of the capsules is enlarged with an increase in the laser exposure time. Jung et al. (2014) reported a nGO -hyaluronic acid composite with spherical morphology and lateral size of ∼250 nm for photothermal ablation therapy of melanoma skin cancer using a near-infrared (NIR) laser. Hyaluronic acid is a widely used transdermal carrier of chemical drugs and biopharmaceuticals. In this work, transdermal delivery of NGO-HA through the normal and cancerous skin was investigated by confocal laser scanning microscopy after labeling the system with a fluorescent dye, Hilyte647. Confocal laser scanning microscopy revealed a negligible (Figure 17) NGO-HA transdermally deliver in healthy tissue and deep penetration into the tumor tissues. The red fluorescence of Hilyte647-labeled NGO-HA was observed in every tissue site including stratum corneum, epidermis, dermis, and tumor tissue, and could be detected from the top and even from the bottom of the dissected 5mm long tumor tissues (Figure 17D). This high penetration resulted both from increased permeability and retention of the tumor mass under the skin and from targeting due to over-expressed hyaluronic acid receptors in the tumor tissue. The same PEG-coated GO nano-system shows lower penetration (Figure 17E). The NIR irradiation (808 nm, 2 W/cm^2^) for 10 min resulted in complete ablation of tumor tissues with no recurrence of tumorigenesis.

**FIGURE 18:**
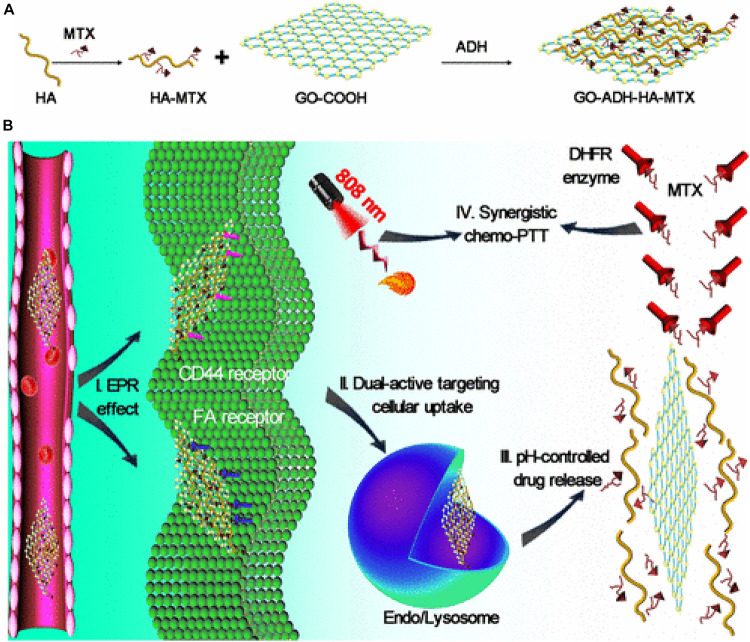
**(A)** synthetic route for GO–ADH–HA–MTX nanosystems; **(B)** schematic illustration of GO–ADH–HA–MTX nanosystems for combination therapy of chemotherapy and PTT through dual-active targeting delivery: (i) accumulation of nanosystems within tumor sites through EPR effect; (ii) improved tumor cellular internalization of nanosystems through dual active targeting mechanisms by specific recognition for HA or MTX; (iii) controlled release of MTX from nanosystems through acid-induced cleavage of ester linkage; (iv) combination therapy of chemotherapy and PTT. Reproduced with permission from Zhang, H. et al. Multifunctional Nanosystem Based on Graphene Oxide for Synergistic Multistage Tumor-Targeting and Combined Chemo-Photothermal Therapy. Mol. Pharm. (2019) doi: 10.1021/acs.molpharmaceut.8b01335. Copyright (2019) American Chemical Society.

The photothermal effect of GO nanoparticles was also investigated by two-photon excitation induced by an ultrafast pulsed laser. Two-photon microscopy with a NIR laser is a hopeful technique for premature detection and therapy of cancer due to the lower background signal, penetration into deep tissue (due to low Rayleigh diffusion and low tissue absorption of NIR light), reduced photobleaching, and reduced phototoxicity. GO nanoparticles with dimensions around 30 nm were prepared and functionalized with transferrin, an efficient ligand for targeting cancer cells that overregulate transferrin receptors, and PEGylate to prolong their circulation in the blood (Li et al., 2012). The two-photon luminescence spectrum of the sample irradiated with a Titanium: Sapphire femtosecond laser, in the range between 400 and 650 nm, had a maximum at 590 nm. The logarithmic scale graph of photoluminescence intensity versus incident power fits a straight line with a slope close to 2, indicating that photoluminescence is due to the excitation of two photons. The photothermal effect was estimated *in vitro* in gastric cancer cells (AGS). After irradiation with a laser power of about 4 mW, the integrity of the cell membrane of the cells incubated with GO became compromised, while an order of magnitude higher laser power was required to induce cell damage or death in the control cells. Also, when the incubated GO cells were raster-scanned at 4 mW, the formation of bubbles (black dots) was instantly noticeable, the addition of ethidium bromide after a couple of minutes revealed staining on most cells. Increasing the laser power to 8 mW it resulted in a more intense perforation of the cell membrane, confined only to the area exposed to laser radiation. The authors attributed the strong micro bubbling observed to the instantaneous heat production by GO particles as a result of laser radiation. The irradiation of an ultrafast pulsed laser can generate a large number of hot carriers with electrons in the conduction bands and holes in the valence bands. The temperature of the hot carriers can increase by a few thousand degrees in about 50 femtoseconds from the absorption of two photons. These hot carriers then recombine releasing energy through photoluminescence emission and through collisions with the GO lattice that causes an increase in temperature. The temperature increase caused by laser excitation should be able to *in situ* reduced GO by producing CO_2_, which increases the formation of bubbles. The collapse of the microbubbles can produce high-pressure shock waves that mechanically disrupt cell membranes and cause instant cell death (necrosis). nGO platforms are widely explored for combined anticancer therapies. Achieving the combination of chemotherapy and photothermal therapy is an important goal for next-generation cancer treatments as it allows a higher death rate of cancer cells with a lower dose of the drug, minimizing side effects and multi-drug resistance. Zhang W. et al. (2011) studied the combined effect of photothermal therapy and chemotherapy of nGO coated with polyethylene glycol *in vitro* and *in vivo* demonstrating that at a fixed laser frequency and concentration of the drug-loaded on the carrier, combined therapy had a superior efficiency in the complete ablation of tumors compared to individual treatments. Qin et al. (2013) explored the chemo-photothermal therapy synergic effect of nGO coated with polyvinylpyrrolidone and functionalized with folic acid. The system showed an ultrahigh loading ratio of DOX, and extraordinary photothermal energy conversion efficiency when irradiated with 2W/cm^2^ NIR laser at 808 nm. In elegant work, Zhang H. et al. (2019) presented a multifunctional nanosystem based on GO for synergistic multistage tumor-targeting and combined chemo-photothermal therapy. First the authors synthesized a prodrug consisting of hyaluronic acid and methotrexate (MTX) via an esterification reaction. MTX is a chemotherapy agent structurally similar to folic acid and able to achieve effective cell internalization through specific interactions with FA receptors over-expressed by various tumor cells. The MTX-HA prodrug was grafted to carboxyl-functionalized GO (GO-COOH) through a relatively stable adipicdihydrazide (ADH) cross-linker via an amidation reaction (Figure 18A) that ensures nanosystem stability during blood circulation after intravenous injection. As described in Figure 18B the accumulation in tumor tissue and within cancer cells was achieved through the MTX-HA dual active targeting mechanism. After cell internalization, the ester bond between MTX and HA was cleaved in acidic endo/lysosomes inside tumor cells (pH 4.5-5.5) (Li Y. et al., 2018) to achieve MTX release, and simultaneously the high local heat-induced by NIR radiation on the GO platform leads to highly efficient cell death via the combination of chemotherapy and PTT therapy.

The intrinsic photoluminescence of nanosized GO and the unique photostability make this material also applicable in the field of biomedical imaging. Contrast agent-based imaging techniques are used for the early detection of tumors, for understanding the distribution of the nanocarrier in tumors and other organs, for evaluating the therapeutic efficacy, and for post-treatment monitoring (Stankovich et al., 2007). Sun et al. (2008) proposed GO nanosheets with lateral dimensions below 10 nm, obtained by a density gradient ultracentrifugation method used for live cell imaging in the near-infrared (NIR). In this work, nGO was coated with polyethylene glycol and covalently conjugated with a specific Rituxan B-cell antibody (anti-CD20) for selective targeting of B-cell lymphoma cells (nGO-PEG-Rituxan). NIR fluorescence images showed that the nGO-PEG-Rituxan conjugate was effectively internalized and exhibited luminescence in the visible and NIR regions, although the quantum fluorescence yield was difficult to quantify due to the inhomogeneous species in the sample. The system was also assessed as an anticancer nanocarrier, by loading DOX through non-covalent interactions π-stacking, demonstrating a high loading capacity and selective inhibition of cell growth. Kalluru et al. (2016) functionalized a GO with lateral size less than 100 nm with NH_2_-PEG-folate moiety via carbodiimide crosslinker chemistry to form GO-PEG-folate to ensure the targeting ability of the folate moieties to the folate receptors on cancer cells. This system exhibited wavelength-dependent single-photon excitation-induced photoluminescence in the short NIR and visible region and acted as a single-photon excitation induced *in vitro* fluorescent cellular marker. Moreover, the authors demonstrated, for the first time, that nGO-PEG-folate can induce singlet (Bray et al., 2018) O_2_ generation upon NIR light excitation. This finding is particularly relevant in the field of cancer therapy because it allows considering GO not only as a photothermal agent but also as a photodynamic agent. Photodynamic therapy (PDT) is Food and Drug Administration (FDA) approved treatment for a variety of oncological, cardiovascular, dermatological, and ophthalmic diseases. PDT utilizes molecule or nanoparticle, named photosensitizer, to convert oxygen from the triplet ground state to the singlet state. This highly reactive oxygen species react with cellular components such as DNA or proteins and do irreversible damage to their structure or function causing apoptosis (Aioub et al., 2018). Until this study, GO was considered as a platform for the loading and delivery of photosensitizer for PDT, which, in most cases, are hydrophobic in nature (Zhou et al., 2012; Zhou L. et al., 2014). In this work, the generation of ^1^O_2_ by the photo-excitation of GO-PEG-folate was investigated with fluorescence lifetime spectroscopy upon excitation using 808 nm and 980 nm light excitation, a ^1^O_2_ phosphorescence emission at ∼1265 nm was detected only upon 980 nm excitation. The photodynamic effect was demonstrated *in vitro* on GO-PEG-folate internalized B16F0 melanoma cancer cells, after photo-irradiation using 808 and 980 nm lasers high intracellular ROS levels and a high percentage of cellular deaths were observed. The percentage of cellular deaths by 980 nm light irradiation was about 1.9 times higher than the cellular deaths observed using 808 nm light, as GO-PEG-folate can sensitize generation of ^1^O_2_ only upon 980 nm light, the cellular deaths observed at 808 nm excitation was probably due to the PTT and PDT combination. The auto-fluorescence of biological tissue may interfere with the intrinsic visible fluorescence of nGO limiting the application of this material in classical imaging techniques (Yang et al., 2013). However, materials with a photothermal effect usually can be used as contrast agents for photoacoustic imaging, (PAI) an innovative, hybrid, non-invasive imaging method that combines the advantages of optical and ultrasound methods. (Yang et al., 2007) is based on the detection of ultrasound waves produced by the photothermal expansion of light-absorbing tissues or contrast probes under pulsed laser irradiation and has attracted broad attention as an exciting non-invasive imaging method for tumor imaging and monitoring of tumor angiogenesis because it allows an unprecedented sensitivity (Cook et al., 2013) and it is not affected by the autofluorescence in biological tissues commonly experienced by fluorescence imaging. The majority of GO PAI imaging studies exploit the large surface area of carbon atoms and the variety of oxygenated functional groups to covalently or non-covalently bind organic or inorganic contrast agents and thus increase NIR absorption and PAI signal (Wang et al., 2013; Yan et al., 2015; Rong et al., 2016). Very recently Jun et al. (2020) presented the results of a derivative of GO as a theranostic agent for photoacoustic imaging-driven tumor-targeted photothermal therapy. The GO nanosheets used in this work were functionalized with folic acid, (FA), conjugated chitosan, (CS), through physical adsorption via hydrogen bonding and electrostatic interaction. The resulting derivate presented a lateral dimension of around 400 nm, showed the ability to target folate receptors, low toxicity, even at high concentrations, and was proved to be an excellent photothermal agent able to destroy more than 80% of cancer cells under laser irradiation. The ability of FA-CS-GO to function as a new PAI contrast agent was demonstrated *in vivo* performing PAI imaging before and after FA-CS-GO injection via tail vein in mouse tumor-bearing. Before injection, only major blood vessels (with hemoglobin as an endogenous contrast agent) in the tumor area are observed. After 24 h from the injection of FA-CS-GO, instead, the PA signals in the tumor area are higher and an outline tumor microstructure was observed, suggesting the gradual accumulation of FA-CS-GO in the tumor area due to the receptor-mediated endocytosis.

## Conclusion

Nanotechnology has been recognized in 2006 by the National Cancer Institute as the science that can effectively change the basis for the diagnosis, treatment, and prevention of cancer, allowing the study and treatment of this disease at a molecular scale, in real-time and during the early stages of the process. 2D nanomaterials possess special physicochemical properties (e.g., light, ultrasonic and magnetic responses) and biological behaviors such as endocytosis, biodistribution, biodegradation, and excretory pathways, which lead to their use in various biomedical applications. In particular, among 2D nanomaterials, graphene and its derivatives have attracted enormous attention in cancer diagnosis and therapy because they combine, in a unique material, extremely small size, NIR absorption, delocalized electrons, extremely high surface area, and versatile surface functionality. GO, initially considered as an intermediate of one of the graphene production processes, has become a material that can be considered both for fundamental research and for its potential applications. GO is one of the most explored materials in nanomedicine due to its extraordinary intrinsic properties. The GO has a high surface area that can be exploited for the loading of drugs and biomolecules, forms stable colloidal dispersions in water and its structure contains a variety of oxygenated functional groups useful for the covalent modification. Furthermore, GO has interesting optical properties useful for phototherapy and biomedical imaging. Known since the beginning of the last century, the oxidation of graphite with strong oxidants in concentrated acid media remains the most commonly used method to produce GO on a large scale. However, the lateral dimensions of GO prepared by this convenient method have a very polydisperse distribution in the range of tens of nanometers to hundreds of micrometers. Obtaining GO sheets with narrow size distribution in the nanometer range is very desirable especially for the nascent biomedical applications of GO. As is known, the physicochemical properties of the material, in particular the lateral dimensions, influence both the optical properties and the absorption and cellular response. Therefore, size is one of the first factors to check when considering GO as a therapeutic platform. Considering the importance of reducing the lateral size of GO nanosheet below 50 nm, in this review, we summarize the main methods employed to reduce and homogenize in nanometric scale the lateral dimensions of GO produced by chemical exfoliation of graphite. For this purpose, a variety of methods have been developed which can generally be distinguished in direct controllable synthesis and post-synthesis separation. The direct controllable synthesis approach involves intervention during the chemical oxidation process by using different precursors, or by tuning the reaction parameters (amount of oxidants, time, and temperature) o by employing physical promoters which mechanically break pristine graphite or GO sheets obtained. The post-synthesis separation involves instead the GO bulk fractionation through centrifugation or selective precipitation in organic solvents, or different pH aqueous solution. nGO platforms are widely explored for combined anticancer therapies. Indeed, together with the ability to accumulate a large amount of drug, for high surface development, they can be photo-stimulated producing heat and CO_2_, which both induce cell death. Moreover, they can be detected for their photo-luminescence properties providing the possibility to have a theranostic tool. All these features represent an important goal for next-generation cancer treatments as they allow a controlled and higher death rate of cancer cells with a lower dose of the drug, minimizing side effects and multi-drug resistance.

## Author Contributions

IT and RV wrote the manuscript. RV and PN revised the manuscript. All authors did the conceptualization contributed to the article and approved the submitted version.

## Conflict of Interest

The authors declare that the research was conducted in the absence of any commercial or financial relationships that could be construed as a potential conflict of interest.
